# Intelligent Generation Method and Sustainable Application of Road Systems in Urban Green Spaces: Taking Jiangnan Gardens as an Example

**DOI:** 10.3390/ijerph20043158

**Published:** 2023-02-10

**Authors:** Lina Yan, Yile Chen, Liang Zheng, Yi Zhang, Xiao Liang, Chun Zhu

**Affiliations:** 1 Faculty of Humanities and Arts, Macau University of Science and Technology, Taipa, Macau 999078, China; 2 Shanghai Total Architectural Design & Urban Planning Co., Ltd., Shanghai 200433, China; 3 Shanghai GOODLINKS International Design Group, Shanghai 200051, China; 4 Shanghai Pudong Architectural Design & Research Institute Co., Ltd., Shanghai 200120, China

**Keywords:** urban green space, road intelligent generation, design method, parameterization, genetic algorithm

## Abstract

This paper takes the garden road system as the research object and proposes a method of generating paths for classical gardens based on parametric design. Firstly, by studying the distribution characteristics of roads, the data on the curvature, angle, and view area of roads were collected. Secondly, the obtained data were transferred to the parameterized platform, and a method of intelligent generation was used for calculation. Finally, the road system was optimized by the genetic algorithm for better application in modern landscape design. According to the current situation, the road system plan generated by the algorithm inherits the characteristics of classical garden roads. This method can be applied to the courtyard, the community park, the urban park, and other objects. This research not only identifies the characteristics of landscape cultural heritage, but also produces an innovative, intelligent design tool. It provides new methods for the parameterized inheritance and application of traditional landscape heritage.

## 1. Introduction

### 1.1. Research Background

With a global emphasis on ecological and cultural landscapes, urban parks, and national parks, a series of modern gardening movements have followed in areas as large as scenic spots and national parks, and as small as pocket parks by the street and green spaces in the corners of residential areas. China also attaches great importance to the construction of the urban environment and seeks an urban landscape that has its own characteristics and is suitable for contemporary and future urban development. More people are realizing that traditional gardens need to be incorporated into the construction of contemporary urban spaces as a part of the urban area’s characteristics. Garden expositions at certain celebrations, the community green spaces of overseas Chinese, and antique-style modern gardens can be seen everywhere. In terms of city image, there is a lack of understanding of traditional gardens, and the construction of a large number of antique parks neither incorporated nor conveyed traditional characteristics, destroying the public’s impression of traditional spaces. Chinese classical gardens have their own characteristics. In particular, the idea of Jiangnan gardens that create natural forests in the city has been passed on from past generations. Facing the continued development of the urban landscape today and in the future, our concern is how to capture the design essence of traditional gardens in a way that is suitable for contemporary and future needs.

Gardens are not spaces that exist alone, but are an important part of the urban space system. Among them, the garden road system is an important element of classical gardens. It functions to organize traffic, connect garden landscape elements, divide garden tour space, and guide tour routes. It can even be said that the planning and design of their garden roads are the essences of the classical gardens and the focus of contemporary landscape design planning.

Therefore, we want to identify the characteristics of traditional garden roads by studying the garden road system and then find a way to capture the characteristics of these traditional spaces to make the past serve the present, utilize traditional and excellent design ideas in advanced and modern ways, and adapt these characteristics to current spatial development needs and future scenarios.

### 1.2. Literature Review

#### 1.2.1. Analysis of Design Methods of Sustainable Urban Green Space Landscape

At present, urban green spaces mainly include comprehensive parks, community parks, specialized parks, strip parks, and street green spaces, including any water areas within their boundaries. However, the term does not include subsidiary green land, production green land, protected green land, or other green land. In addition, there are small green spaces in buildings for public use that can also be classified as urban public green spaces. In the past, traditional urban green space design was carried out by landscape architects during on-site surveys, and the design was completed in combination with the needs of society or of the property owners. Presently, common urban green space design methods mainly include the following: using virtual reality (VR) technology to obtain user and expert opinions for the design of green business parks (GBPs) [1]; urban green space design that responds to human needs for recreation, amenities, and environmental quality [2,3,4,5]; the optimal planning of an urban greening system for high-density cities in response to the urban microenvironment based on a genetic algorithm [6]; a method for planning urban green spaces that combines natural solutions and GIS tools [7]; medicinal landscape design based on the theory of the five senses [8]; and urban green space design methods based on disease prevention or suppression [9,10,11,12,13,14,15].

#### 1.2.2. Method and Model Analysis of Road Organization in Jiangnan Classical Gardens

However, with the advancement of modern gardening in China in this century, we have begun to explore the methods and models of Jiangnan classical garden space creation and garden road organization. The Craft of Gardens, Jiangnan Garden Chronicles, Suzhou Classical Gardens, and Jiangnan Gardens, the classic works of Chinese gardening, all mention the study of garden paths [16,17].

In modern times, most of the research has been based on text description, and there have been few quantitative research studies. For example, some scholars used “cognitive imagery” to explore the visual perception of the Lingering Garden in space [18]. Another study used the theory of cognitive narratology to explore the influence of paths in private gardens such as the Garden of Cultivation, the Lingering Garden, and the Master of the Nets Garden in Suzhou, which were established during the Ming and Qing Dynasties [19]. The spatial sequence of the entrance of the Lingering Garden was analyzed with the viewshed analysis theory [20]. The path space of private gardens in the Jiangnan region was studied by using the combination of hierarchical classification analysis, modulus theory, “picture-bottom” theory of Gestalt psychology, and topology theory [21]. In addition, some scholars analyzed garden area, path length, path projection length, and morphological change according to Chinese and Western gardens’ winding-path patterns, and obtained the design methods’ rules [22].

In recent years, scholars have applied advanced ideas and techniques to road research. Research addressing quantification, empirical video analysis technology, geographic information data, and other aspects of the field has increased. For example, the spatial layout and path relationship in the Humble Administrator’s Garden, the Lingering Garden, and the Master of the Nets Garden were determined with quantitative indicators including static line of sight, sight distance, visual field, dynamic path spacing, and scene enclosure [23]. The stagnant point research method based on video analysis technology was used to empirically analyze the stagnation point law of the Master of the Nets Garden and the Lingering Garden [24,25]. Another study carried out the simulation and application of the urban road landscape based on geographic information data [26]. Further, research has been conducted on algorithms and the architecture of software systems for automated natural and anthropogenic landscape generation [27].

#### 1.2.3. Research Frontier Analysis of the Intelligent Design Method

Parametric design is a method of intelligent design that uses parameters to describe the variables to realize the repeatability and adjustability of the design [28]. Although parameter manipulation in parametric design cannot be strictly regarded as a design, the design solution is defined as a controlled design application through parameter scripts and algorithms, so that the parametric design has the variability and fixity of general design behavior [29]. In recent years, parametric design has been widely used. In the field of construction decoration, a series of parametric design studies on well-known buildings and building components have emerged [30,31,32]. In the field of architectural sustainability, parametric design is often combined with physical environment simulation technology to derive parametric design through environmental and climate parameters [33,34,35]. In the field of landscape design, under the influence of complexity theory, the characteristics of complexity and diversity and the method of parametric design have played an important role [36]. Traditional landscape design often relies on the subjective and empirical analysis and judgment of professionals. With the popularity of parametric design, many researchers began to conduct parametric design research on complex elements in landscape design [37]. The traditional design methods of Jiangnan gardens are often expressed by poetic words in classical literature, and there are no specific design parameters for designers to refer to, which makes it difficult for modern designers to design Jiangnan garden-style landscapes. In existing studies, researchers have conducted quantitative research on the natural and environmental characteristics of Chinese classical gardens [38]. However, the application of the parametric design method to Chinese classical gardens still needs to be explored, because it will not only help landscape architects to improve design efficiency, but also help to preserve the characteristics of classical gardens.

### 1.3. Problem Statement and Objectives

On the whole, although the method of road organization is complex, scholars have entered into quantitative exploration, and the corresponding indicators and path design rules have achieved certain results. However, considering the visual space perception of “moving and changing scenery” in classical gardens and the more challenging continuity design of traditional garden characteristics, this area of research still needs to be developed and studied. Artificial intelligence and parametric design are popular research topics at present. Parameterized and algorithmic intelligent generation of garden road system research has great exploration value for the present and future.

Tracing it back to the source, one of the keys to solving the problem also happens to include capturing the characteristics of classical gardens, perceiving the essence of garden paths, and applying traditional wisdom to modern landscape design. To this end, by studying the distribution characteristics of roads in traditional gardens (corresponding to the Hamiltonian cycle) and their tortuosity (curvature of road), the characteristic data of traditional gardens and roads represented by Jiangnan gardens were collected. At the same time, the obtained data were transferred to the parameterized platform, and a method of intelligent generation was used for calculations. Finally, the “wisdom of design” was realized through the application of drawing modern landscape designs through genetic algorithms. In this paper, the researchers explored the following four questions:

(1) What are the distribution characteristics of traditional garden roads in Jiangnan classical gardens that can help implement intelligent garden road design?
(2) How can we combine the collected road curvature, angle, and field of view data into the algorithm model?
(3) How does the genetic algorithm assist in the intelligent design of garden roads?
(4) How does this method work when it is used to build courtyards, community parks, and urban parks?

## 2. Materials and Methods

### 2.1. Method Process

**Figure 1:**
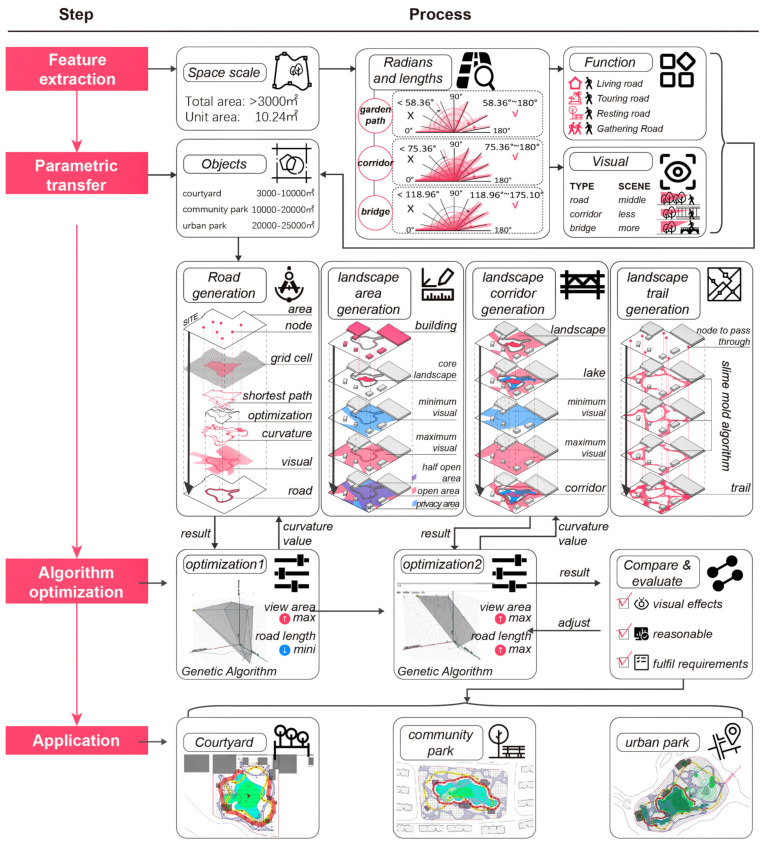
Method flow diagram.

This paper proposes a method, based on parametric design, to realize the generation of outdoor paths from the site outline and basic elements of the Jiangnan classical garden style. Additionally, it provides applications for the renovation, restoration, upgrading, design, and other aspects of garden projects focusing on the traditional Chinese style (Figure 1). Specifically, these findings can be used in courtyards, community parks, urban parks, and other spaces, and have important practical value for the unification of regional styles in garden design and the protection of garden features.

The method of this study consists of four aspects: feature acquisition, parametric translation, algorithm optimization, and model application. The details are as follows (Figure 1):

(1) Feature extraction: First, it is necessary to clarify the specifications and characteristics of Jiangnan classical gardens. In this study, based on previous statistics, the area of Jiangnan classical gardens is taken to be larger than 3000 square meters, and the grid is divided into portions of 10.24 square meters (3.2 m × 3.2 m) as area units (according to the traditionally constructed measurements) (In the construction measurements of the Ming and Qing Dynasties, one *chi* = 32 cm and one *zhang* = 3.2 m). Secondly, the roads in Jiangnan classical gardens can be divided into three types: roads, corridors, and bridges. According to the records from The Craft of Gardens, the curvatures of different types of roads have different rules [39]. All roads in the Jiangnan classical gardens are counted, and their curvature is converted into radians to be utilized as one of the constraints and generation rules of the parametric design. Finally, the study found that the roads in Jiangnan classical gardens have various uses, including daily life functions, sightseeing functions, rest functions, and gathering functions, and these functions are mainly related to the spatial scale and field of view of the garden. The view of the bridge is the widest, the view of the road is the second widest, and the view of the corridor is the smallest.

(2) Parametric transfer: The parametric design uses Rhino’s Grasshopper as the software platform. Grasshopper is one of the most commonly used parametric software platforms. It can be utilized for visual programming, and it is widely used in the field of design and analysis [40]. According to the characteristics of classical gardens and the needs of modern landscape design applications, the application’s targets are positioned as three types: courtyards (3000 to 10,000 m^2^), community parks (10,000 to 20,000 m^2^), and urban parks (20,000 to 25,000 m^2^). From these three types of applications, the base elements for parametric design are extracted, such as site boundaries, points of interest, existing buildings, and lakes. The translation process is mainly divided into road generation, landscape area generation, landscape gallery generation, and landscape trail generation.
  ① In road generation, the shortest path is generated according to the site boundary and points of interest. The path divides the site into a network according to the grid cell (10.24 m^2^/cell) of the Jiangnan classical garden, uses the Hamiltonian cycle [41] to generate a new path, optimizes the path according to the known curvature, and, finally, further adjusts the design of the obtained road according to the viewshed analysis.
  ② In the generation of the landscape area, firstly, the core landscape area is generated with the inherent buildings and the above-generated roads as constraints, and, secondly, the view area is analyzed for the path of the road to obtain the minimum and maximum view areas. The two fields of view are superimposed, and finally, a semi-open landscape area, an open landscape area, and a private landscape area are obtained.
  ③ In the generation of the landscape gallery, according to the generated road and landscape area, the building and lake elements are imported at the same time. The road segment where the lake and the road intersect will generate a bridge; then, the viewshed analysis is performed around the lake and the road, and the maximum boundary of the overlapped viewshed area will generate a corridor. In addition, the corridors of Jiangnan classical gardens are usually not a closed loop (only about half), and the paths of some corridors can be deleted according to the actual situation of the site.
  ④ In the generation of landscape trails, there are usually many landscape features that adorn the landscape space in Jiangnan classical gardens, such as stacked rocks, rockeries, precious plants, etc. [42] The number of these landscape features is determined according to the financial resources of the garden owner. Landscape features can be used as necessary points in landscape trails, and landscape trails can be generated with the slime mold algorithm. The slime mold algorithm is one of the optimization algorithms which simulates the spreading and foraging behavior of slime mold, and is also commonly used in the field of simulating biological behavior to find the optimal path [43]. By simulating the slime mold algorithm three times, the simulation results can be superimposed on each other, and finally, the landscape trail is generated.

(3) Algorithm optimization. In order to further make the results generated by the parametric design conform to the characteristics of Jiangnan classical gardens, the roads and corridors from the above-generated results are optimized via a genetic algorithm. The genetic algorithm is one of the algorithms for solving unconstrained and constrained nonlinear optimization, one which can effectively solve the problem of global optimization [44].
  ① In the optimization of the roads, the genetic algorithm is used to find the combination of the maximum field of view and the minimum path length based on the radian of the road, with Jiangnan classical gardens as the optimization result.
  ② In the optimization of the corridors, the genetic algorithm is used to find the combination of the maximum field of view and the maximum path length based on the radian of the road, with the Jiangnan classical garden as the optimization result.
  ③ An artificial judgment is made on all the above-generated results, as the numerical optimal result of the parametric design may not be the optimal design result. The principles of judgment: Did the effect of the visual field achieve the expected goal? Is the overall layout reasonable? Can the final design meet the needs of the designer, party A, and the design specification, such as the road network density required by the design specification? If the results do not meet the above principles, they can be adjusted again.
(4) Model application: According to the above process, the model can be applied to courtyards (3000 to 10,000 m^2^), community parks (10,000 to 20,000 m^2^), and urban parks (20,000 to 25,000 m^2^). The differences between the three are not only reflected in the scale of the area, but also in their inherent buildings, water bodies, points of interest, and must-pass points. The model can be well adapted to the differences between these elements and has practical value in both new landscape design and old landscape renovation.

### 2.2. Analysis of Path Characteristics

#### 2.2.1. Road Types and Characteristics

**Figure 2:**
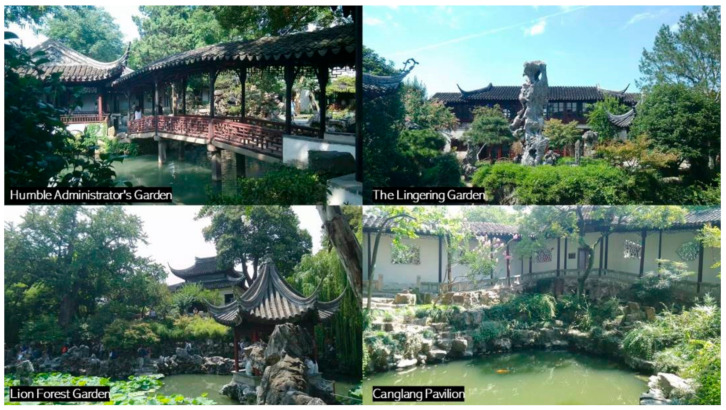
Typical representative images of Jiangnan gardens. (Image source: photographed and annotated by the author).

The Ming and Qing Dynasties were the mature period in the development of Jiangnan classical gardens in China, and when the unique gardening art of Chinese classical gardens was most highly developed. The objects of this study are nine typical classical gardens in the Jiangnan region of China: Humble Administrator’s Garden (The Humble Administrator’s Garden is a UNESCO World Heritage Site and one of the most famous of the gardens of Suzhou.), Lingering Garden (Lingering Garden is a renowned classical Chinese garden, dating back to 1593. It is located at 338 Liuyuan Rd.,. Suzhou, Jiangsu Province, China. Since 1997 it has been recognized with seven other classical gardens of Suzhou as a UNESCO World Heritage Site. The garden also contains two UNESCO Intangible World Heritage Arts: Pingtan and Guqin music.), Canglang Pavilion (The Canglang Pavilion was built in 1044 CE by the Song dynasty poet Su Shunqin (1008–1048) on the site of a pre-existing imperial flower garden c. 960 CE. It is the oldest of the UNESCO gardens in Suzhou, keeping its original Song dynasty layout.), Master of the Nets Garden (Master of the Nets Garden, previously called Ten Thousand Volume Hall, was first constructed in 1140 by Shi Zhengzhi. It is recognized with other classical Suzhou gardens as a UNESCO World Heritage Site.), Garden of Harmony, (The Garden of Harmony, otherwise called Yuyuan, was built in the Qing Dynasty by Gu Wenbin, an official from the Qing Dynasty.) Lion Forest Garden (The Lion Grove Garden is a garden located at 23 Yuanlin Road in the Gusu District (formerly Pingjiang District), Suzhou, Jiangsu, China. The garden is famous for the large and labyrinthine grotto of Taihu rocks at its center.), Retreat and Reflection Garden (The Retreat and Reflection Garden is a notable classical garden in China.), Couple’s Retreat Garden, and Jichang Garden is a famed Chinese classical garden, and it was claimed as a nationally protected location of historical and cultural relics on 13 January 1988.), which were selected for special analysis of their internal roads (Figure 2).

“The scenery is different when you walk, and the winding path leading to the secluded” is a typical feature of the paths of Jiangnan classical gardens. Since ancient times, gardening in the Jiangnan classical gardens has paid attention to the relationship between architecture and mountains, rivers, and trees, and pursued the creation of natural beauty without artificial traces in the garden landscape. As an important connection between building and landscape, the manner of its reflecting the beauty of nature over the course of a path is particularly important for its layout design.

**Figure 3:**
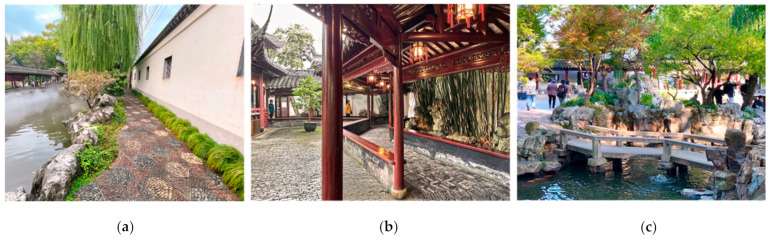
Types of roads in Jiangnan classical gardens: (**a**) garden path; (**b**) corridor; and (**c**) bridge. (Image source: photographed by the author.)

There are various types of roads in Jiangnan classical gardens, consisting of garden paths, corridors, and bridges. Among them, garden paths are the main road-type in landscape gardens. The garden path is located within a landscape of mountains, stones, flowers, and trees, and it is provided as a way for the owner of the garden to enjoy the landscape. The corridor is a unique type of road in Jiangnan classical gardens. It is usually located between the main buildings, connecting them together. The climate in the Jiangnan classical gardens is humid and rainy, and the corridor, as a roofed road, acts as a shelter from wind and rain, ensuring accessibility between buildings that mainly have residential functions. At the same time, the zigzagging of corridors within the landscape can also provide a good viewing experience. The bridge is the water extension in Jiangnan classical gardens, such as can be seen in the roads of the Humble Administrator’s Garden. There are lakes and water systems to the south of the Yangtze River, and the water area in the garden is relatively large. While the bridge is used for water transportation, it becomes a unique landscape presentation in the garden, together with the water (Figure 3).

#### 2.2.2. Path Function and Distribution Characteristics (Spatial Sequence)

Jiangnan classical gardens are built in the city; thus, one question is: how to create a natural landscape suitable for people’s lives in the cramped urban space? This is the same problem found in modern urban landscape construction. In order to create a rich and varied landscape-effect in a limited space, the roads of Jiangnan classical gardens have unique layout characteristics.

**Figure 4:**
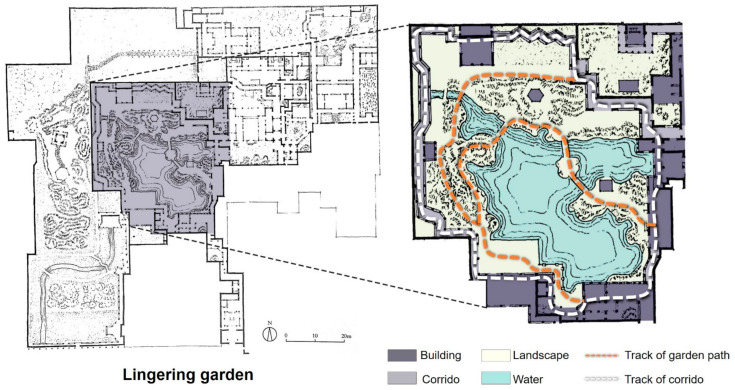
Floor plan of the central part of the Lingering Garden.

The first layout is the “space loop”, in which the road provides accessibility with regard to the residential function. Jiangnan classical gardens usually have a loop through each functional building, connecting each functional area in a series. This loop is connected, in turn, by garden paths or corridors, forming the main road in the garden. For example, the road system in the Lingering Garden seems to be very complex, but from the central part of the paths, it can clearly be seen that the garden roads are arranged in a circular sequence, and the corridors and garden paths each form a spatial loop with the buildings (Figure 4).

The second is the “winding and changing” layout, which mainly provides a tourist function. The layout of the corridor is characterized by winding and twisting, and adopts the method of changing roads. Various types of buildings, such as corridors, pavilions, and waterside pavilions, are arranged around the water’s surface along the perimeter of the garden, forming a visual experience that is centripetal and cohesive. At the same time, the twists and turns of the corridor are added to achieve the effect of changing and continuous scenery. The moving back and forth on the path is tortuous, which invisibly increases the length of the journey and the tour time, and psychologically extends the original sense of space. Walking in the winding and changing corridors and watching the garden scenery on both sides is like looking at a magnificent scroll of Chinese landscapes.

The third is the “degree of twists and turns” layout, which plays a role in enriching pedestrians’ viewing experiences. Appropriate twists and pauses can adjust the walking rhythm between movements, stops, and turns in the process of walking. The corridors and bridges are usually arranged in a “zigzag” shape, although the zigzag angles are usually not rigid right angles. The junction between the corridor and the wall is transformed into a pavilion or landscape of mountains, stones, flowers, and trees that functions as an embellishment and as a node for road rests. For example, the “Moon Come With Breeze Pavilion” in the Master of the Nets Garden not only enriches the experience of the road, but it also breaks the sense of occlusion caused by the flat and rigid walls of the original wall.

### 2.3. Garden Road Curvature and Viewshed Analysis

According to the characteristics of Jiangnan classical garden roads described above, nine typical Jiangnan classical gardens were selected as the research objects for this paper. First, using the two aspects of garden road tortuosity and viewing experience as the starting point, quantitative statistics were collected on the garden roads’ tortuosity and their viewing fields in order to determine the inner law of the garden roads and how they form the feeling of the “winding path leading to secluded, moving with different scenery”. In addition, the statistical results were incorporated into the intelligent generation of road parameters in this paper to form a landscape road system with the characteristics of classical gardens.

#### 2.3.1. Road Analysis Methods

The curvature and angle of the corner directly affect the shape and walking experience of the path. If the characteristic of the garden road is the “winding path leads to secluded”, then the turning curvature and angle of the road are the keys. Therefore, this paper calculated the curvature of garden roads according to three types of garden roads (for the raw statistical data, refer to Table A1 and the summary of nine garden roads in Appendix B): the garden path (refer to Table A2, Table A3, Table A4, Table A5, Table A6, Table A7, Table A8, Table A9 and Table A10 in Appendix B for the raw statistical data), the corridor (refer to Table A11, Table A12, Table A13, Table A14, Table A15, Table A16, Table A17, Table A18 and Table A19 in Appendix B for the raw statistical data), and the bridge (refer to Table A20, Table A21, Table A22, Table A23, Table A24, Table A25, Table A26, Table A27 and Table A28 in Appendix B for the raw statistical data). This method of analyzing the curvature of the road was used to obtain the coordinate values of all turning points from the road’s centerline, and to calculate the curvature and angle of the corners formed by a group of three adjacent coordinated points, thereby obtaining the curvature and turning angle values of the garden road. In addition, visual observation points were set at each corner, and the viewing area of each observation point in the garden was calculated and counted.

#### 2.3.2. Road Curvature Analysis

**Figure 5:**
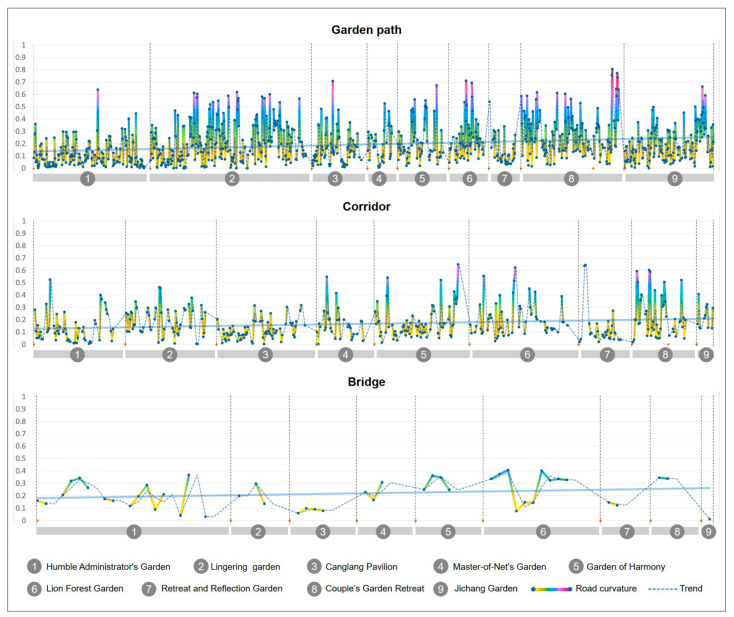
Road curvature analysis map of nine typical gardens.

First, we determined the statistics of curvature, which mainly express the “curvature degree” of the road. By calculating the curvature statistics of the median lines of each road in nine typical gardens, it was found that: the average curvature of garden paths was 0.34, the average curvature of corridors was 0.28, and the average curvature of bridges was 0.20. As far as the overall curvature value is concerned, it can be seen from Figure 5 that the curvature of the garden path had the largest fluctuation range, and its curvature range was 0~0.81 (Figure 5, garden path). The curvature of the corridor ranged from 0 to 0.65 (Figure 5, corridor). The curvature of the bridge ranged from 0 to 0.41 (Figure 5, bridge). It can be seen from the data chart that the tortuous degree of the garden path was the largest, and there were also more bending changes; the tortuous degree of the corridor was relatively stable, and the tortuous degree of the bridge was the smallest, given that there were fewer bending changes.

#### 2.3.3. Road Angle Analysis

**Figure 6:**
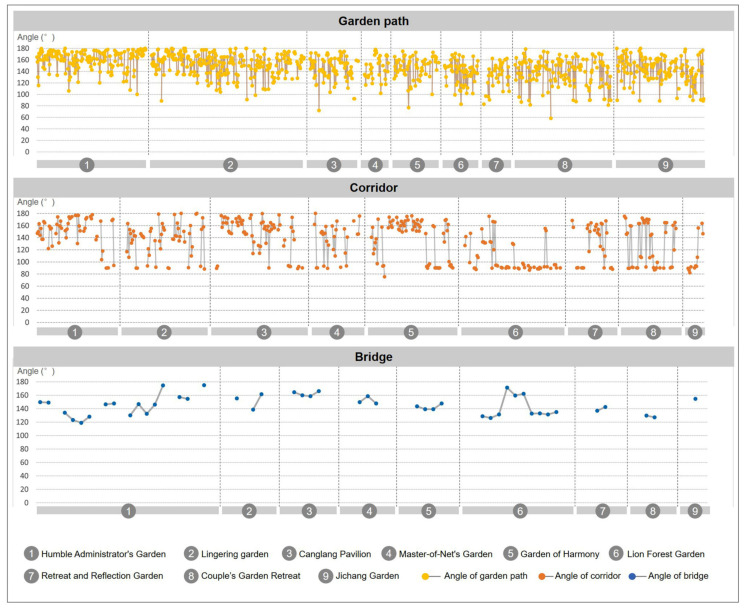
Nine typical garden road system angle analysis diagrams.

The turning angle corresponding to the curvature can more intuitively show the “turning degree” of the garden road system. From the turning angle of the garden road system (Figure 6), it was determined that the turning angle of the garden path was 58.36~180°, the turning angle of the corridor was 75.37~180°, and the turning angle of the bridge was 118.97~175.10°. The range of folding angles of the garden path was large, with the exception of a few relatively sharp angles at 58.36° and 72.13°; most folding angles averaged around 129.40° (Figure 6, garden path). The range of the folding angles of the corridor obeys a certain law, and it can be seen that the frequency of about 85~90° appears at the starting point and the ending point. This shows that almost every section of the corridor will have a small section that follows the edge of the building and presents a phenomenon similar to a right angle. Most of the angles averaged around 130.26° (Figure 6, corridor). The bending angle range of the bridges was relatively small, and the turning angles of all bridges were obtuse, with an average of 150.84° (Figure 6, bridge).

#### 2.3.4. Degree Analysis of Garden Road System Viewsheds

**Figure 7:**
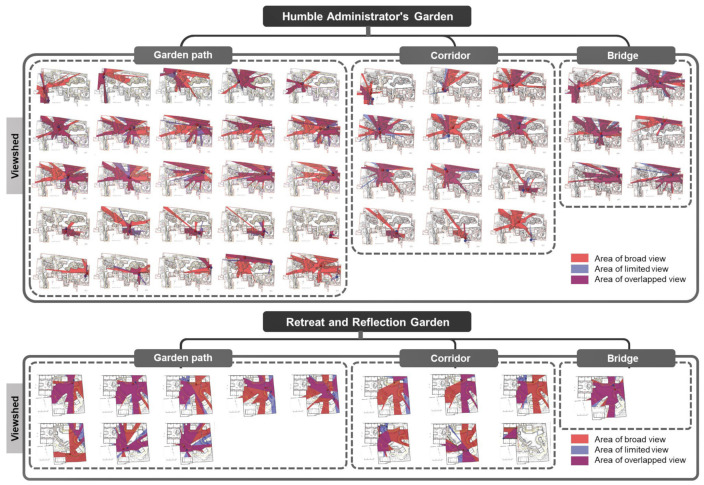
Road viewshed area analysis.

In order to further study the visual perception of the scenery in the process of road walking, we must consider how to reflect the garden road system characteristics of the “moving and changing scenery”. This paper quantified “movement” as the length of the movement during walking, and “view” was quantified as the viewing area at the turning point of the path. Further statistical research was conducted on the turning length and viewing area of each turning point in the process of road-walking (Figure 7).

**Figure 8:**
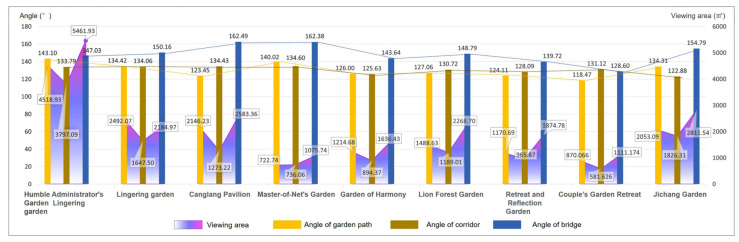
Average angle and average viewing area of road systems in nine Jiangnan classical gardens.

Based on the previous garden road system angle statistics, the researchers analyzed the relationship between the average angle of the overall garden road system and the average viewing area of the nine Jiangnan classical gardens. In Figure 8, it can be clearly seen that the average turning angles of the three road types in the park are relatively close, and the fluctuation trend is small (Figure 8, bar chart). Relatively speaking, the average viewshed area value of each park road varies greatly (Figure 8, area map). Interestingly, the Humble Administrator’s Garden had a high landscape visibility in terms of the total average viewing area, with an average range of about 3797.09 to 5461.93 square meters, while the average viewing area of the rest of the gardens ranged from 965.87 to 1874.78 square meters.

From this analysis, it can be seen that, under the condition of similar angles, the total viewing area of the Humble Administrator’s Garden is much larger than that of the other gardens. Therefore, this study took the Humble Administrator’s Garden as the example of typical paths for the aspect of “moving and changing scenery”, and chose the Retreat and Reflection Garden as the representative garden to carry out a comparative study on the field of view area and road length. Further comparisons were made between the turning unilateral lengths (edge lengths) of various types of garden paths in the Humble Administrator’s Garden and the Retreat and Reflection Garden and the viewing area. Thus, the road length and viewing area charts were formed.

**Figure 9:**
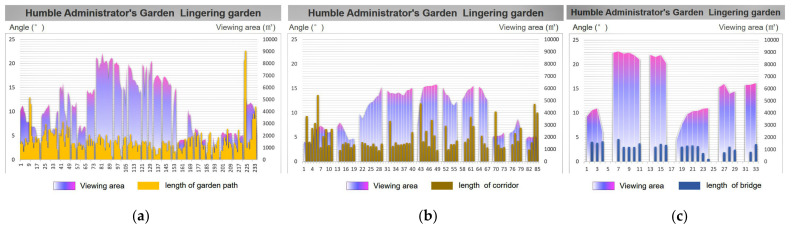
The Humble Administrator’s Garden: overlay map of unilateral length and viewshed area of road turns. (**a**) The garden path length and viewing area; (**b**) the corridor length and viewing area; and (**c**) the bridge length and viewing area.

From the perspective of the turning length of the garden path, the average total length of the garden road in the Humble Administrator’s Garden was 31.21 m, the turning unilateral length was between 0.74 and 22.58 m, and the average viewing area was 4313.55 square meters. The average total length of the corridor was 31.15 m, and its average viewing area was 4017.45 square meters. However, unlike the value of the garden path, the length of the corridor was between 2.26 and 13.60 m. The average total length of the bridge was 11.09 m, the unilateral length of the turning point varied between 0.48 and 4.58 m, and the average viewing area was 6121.33 square meters (Figure 9).

**Figure 10:**
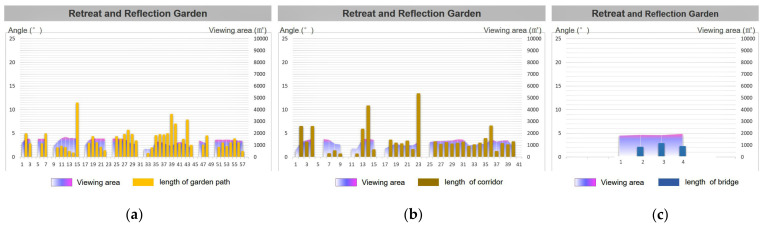
The Retreat and Reflection Garden: overlay map of unilateral length and viewshed area of garden road turns. (**a**) The garden path length and viewing area; (**b**) the corridor length and viewing area; and (**c**) the bridge length and viewing area.

The average total length of the garden path in the Retreat and Reflection Garden was 19.57 m, the turning unilateral length was between 0.79 and 11.46 m, and the average viewing area was 1307.02 square meters. The average total length of the corridor was 28.37 m, the unilateral length of the turning point varied from 0.68 m to 20.94 m, and its average viewing area was 1110.01 square meters. The length of the corridor was about 4~5 m. The average total length of the bridge was 7.38 m, the unilateral length of the turning point varied between 2.15 and 2.91 m, and the average viewing area was 1864.34 square meters (Figure 10).

To summarize, we can represent the relationship between the road turning length and the horizon as follows:

- Garden paths: walk less, see more, and change more. The total walking length of the garden path is short, the turning length changes more, and the visual field changes abundantly.
- Corridor: walk a lot, see a lot, and change a lot. The total walking length of the corridor is long, and the transitions and sights have a certain sense of continuity and rhythm.
- Bridge: walk less, see more, but change less. The total walking length of the bridge is short, the turning length is uniform, and the field of vision is the widest.

Through the above statistical analysis of curvature, angle, and viewing area, this study obtained the characteristics and angle range values of the road turning changes. The angle range value, turning length, and view field law of these garden paths, corridors, and bridges are incorporated into the parametric generation later in this paper as an important basis for affecting the generation of garden road system corners.

## 3. Parametric Construction and Generation Process of a Garden Road System

### 3.1. Algorithms and Generation Process

This paper proposes a method of generating paths for Jiangnan classical gardens based on parametric design. The method is based on the analysis and extraction of path features of nine typical classical gardens, such as path type, function, distribution characteristics, and path radian. At the same time, various methods such as the Hamiltonian cycle, viewshed analysis, and slime mold algorithm were used to derive the path generation of the garden, and the genetic algorithm was used to obtain the globally optimal path design.

The landscape characteristics of Jiangnan classical gardens can be divided into four dimensions, namely, coherence, naturalness, complexity, and historicity [45].

**Figure 11:**
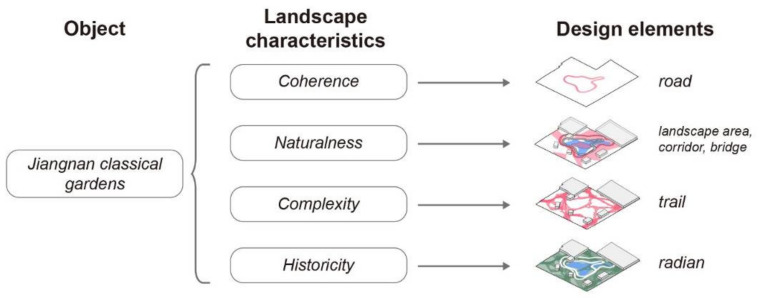
The dimensions of landscape characteristics of Jiangnan classical gardens.

- Coherence is defined as the coherence of space, that is, “space loop”.
- Naturality emphasizes the interaction and connection between people and natural elements (lakes, plants).
- Complexity enriches the form of landscape elements.
- Historicity emphasizes the traditional gardening rules of Jiangnan classical gardens.
- The dimensions of the above landscape features can be translated into parameterized parameters: roads, landscape areas, corridors, bridges, trails, radians, etc. (Figure 11).

(1) For the embodiment of the coherence dimension of Jiangnan classical gardens, the method of the Hamiltonian cycle was adopted in the parametric design.

**Figure 12:**
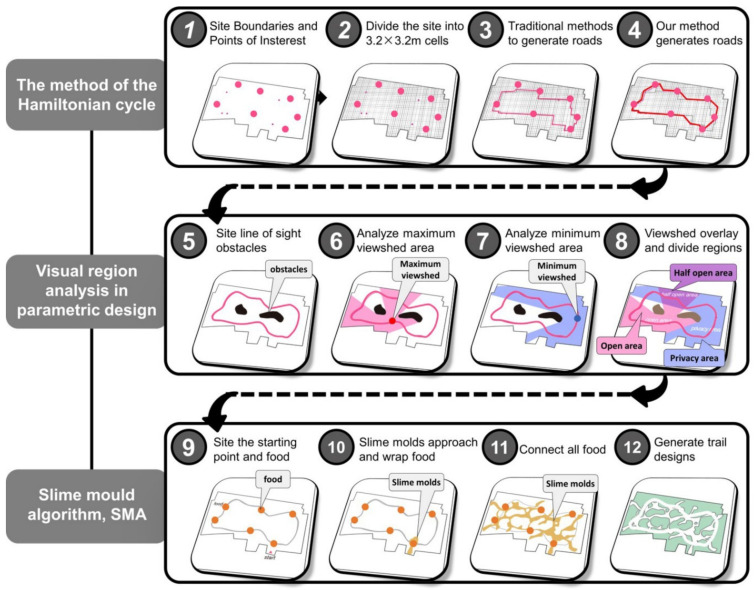
Design method and process (4) For the embodiment of the historical dimension of Jiangnan classical gardens, the genetic algorithm method is mainly used in parametric design. As seen in the prototype of the scheme generated in the process described above, the curvature and viewshed values obtained from the analysis are incorporated into the genetic algorithm to further optimize the path. At the same time, it is also necessary to control the density of the road network and appropriately increase or decrease the length of the path to meet the requirements of the design specifications. The curvature and field of view are obtained from the statistics of the existing Jiangnan classical gardens, and are also very important components of the Jiangnan classical gardens. The relevant laws have continued to exist throughout history, and the new scheme conforms to them, reflecting the historical dimension of the garden.

Specifically:

- Set up points of interest in the site, which can be pavilions, platforms, and buildings in Jiangnan classical gardens (Figure 12(1)).
- Divide the site into several 3.2 m × 3.2 m grids to generate an undirected graph (Figure 12(2)).
- Then, use an algorithm to concatenate these points of interest along the grid lines (Figure 12(3)). The traditional series connection method will cause some roads to overlap and have too many long and straight lines, which is not in line with the characteristics of Jiangnan classical gardens.
- Our method is based on the Hamiltonian cycle and adds new rules: roads cannot overlap and polylines are maximized (Figure 12(4)).
- This method can automatically generate more complex and coherent road loops for the prototype of the road system according to the set parameters.

(2) To reflect the natural dimension of the Jiangnan classical gardens, the visual area is used as the main judgment basis in the parametric design.

Specifically:

- Place sight-blocking objects in the site, which can be rockeries, rocks, and plants (Figure 12(5)).
- Analyze the field of view of the site and calculate the position and area of the largest field of view in the road as an open space (Figure 12(6)).
- Further, carry out the analysis of the field of view, and calculate the position and area of the minimum field of view in the road as a private space (Figure 12(7)).
- Superimpose the above analysis results on the visual field. The overlapping part is set as semi-open space, and the rest is open space and private space (Figure 12(8)).

Different space attributes can be set with different landscapes to help reasonably plan the activity space. For example, set up more event facilities (porches and bridges) in open spaces. In private spaces, a richer natural landscape can be set, and the semi-open space is a compromise between man-made objects and natural objects.

(3) To reflect the complexity dimension of Jiangnan classical gardens, the slime mold algorithm (SMA) method was used in the parametric design. The slime mold algorithm simulates the behavior and morphological changes of Physarum polycephalum during the foraging process. It can ensure the diversification and efficiency of the path, achieving balance between the two [46].

Specifically:

- Set the starting point and food locations in the venue (Figure 12(9)). The starting point is the entrance to the site. The trail acts as an extension of the main road and is therefore set on the main road.
- Start the slime mold so that the slime mold grows from the starting point and forms a path through processes such as covering food locations, extending outward, and path oscillation (Figure 12(10)).
- After a period of time, the slime mold will connect all the food locations in the field, and the changing shape will gradually become stable (Figure 12(11)).
- Stop the growth of the slime mold (Figure 12(12)).

The growth trajectory of slime molds can be used as a reference for the design of garden trails. Each activation of the slime mold yields different results, allowing for different trail designs to be derived. This bionic design derivation process reflects the complex dimension of Jiangnan classical gardens.

### 3.2. Generation Process

The process of scheme generation was implemented via the Grasshopper visual programming application in Rhinoceros 3D modeling software to set various parameters and formulate generation rules through programming in order to realize the final generation of the scheme (refer to Appendix A: Grasshopper environment configuration).

**Figure 13:**
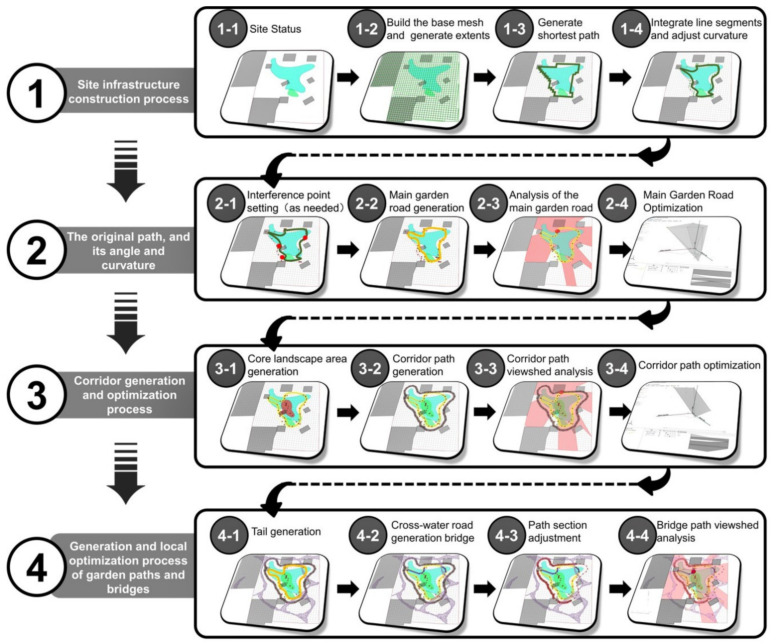
Parametric design steps and procedures.

(1) Site infrastructure construction (Figure 13(1)):

Set the basic conditions of the site, delineate the boundaries of the site, and put the buildings and water bodies that need to be preserved in the current situation into the model (Figure 13(1-1)).

Establish the basic grid (the size of each grid is 3.2 m × 3.2 m) and the generated range (Figure 13(1-2)).

Generate the shortest path by means of the Hamiltonian cycle (Figure 13(1-3)).

Classify the polylines and long straight lines of the shortest path, make the polylines generate smooth curves, and adjust the curvature of the curves (Figure 13(1-4)).

Finally, integrate straight lines and curves as the prototype of the main garden road.

(2) Main garden road generation and optimization (Figure 13(2)):

- In Jiangnan classical gardens, the garden road will be adjusted according to the special features in the garden so that the road is closer or farther away from the features, allowing you to set interference points to fine-tune the garden road (Figure 13(2-1)).
- According to the width of the main road, make the line segment generate the road segment (Figure 13(2-2)).
- Perform a viewshed analysis on the generated main road, recording the maximum viewpoint, the minimum viewpoint, and the viewport area as the parameters of the subsequent design (Figure 13(2-3)).
- Use the genetic algorithm to optimize the main garden road (Figure 13(2-4)).

The curvature value of the main road is controlled, and parameters such as the viewing area and path length are used as target values so that the genetic algorithm can obtain the final curvature value under the condition that the viewing area is the largest and the path length is the smallest through continuous iteration. At the same time, this value also needs to meet the curvature range of Jiangnan classical gardens.

(3) Corridor generation and optimization (Figure 13(3)):

- Derive the scope of the center of the visual field from the path of the main garden road as the area for generating the core landscape (Figure 13(3-1)).
- According to the distance between the corridor and the road in the Jiangnan classical garden, the path offset by the main garden road generates the corridor path (Figure 13(3-2)).
- Perform viewshed analysis on the path of the corridor, recording the maximum viewpoint, the minimum viewpoint, and the view area as the parameters of subsequent design (Figure 13(3-3)).
- Use the genetic algorithm to optimize the corridor’s path. Control the curvature value of the corridor path and access the viewpoint area and path length parameters as the target values (Figure 13(3-4)).

Let the genetic algorithm obtain the final curvature value under the conditions of the largest viewing area and the largest path length through continuous iteration. At the same time, the value also needs to meet the curvature range of Jiangnan classical gardens.

(4) Generation and local optimization of garden paths and bridges (Figure 13(4)):

- Set the site entrance as the starting point, and the buildings and main road nodes in the site as food locations. Then, activate the slime mold algorithm so that the slime mold covers all food locations in the field. The spawning trajectories of slime molds act as a scheme for garden paths. The slime mold algorithm can be restarted continuously to generate different scenarios, and the appropriate one can be chosen as the final garden path (Figure 13(4-1)).
- Set the road across the water as a bridge (Figure 13(4-2)).
- Partial adjustments are made to the path of the corridor, and the corridor can be increased or decreased according to the project cost of the garden road and the needs of landscape zoning. The corridors in the Jiangnan classical gardens are usually not a complete path, and about half of the closed-loop path can be cut (Figure 13(4-3)).
- Perform visual field analysis on the path of the bridge to see if the visual field of the bridge can cover important landscape areas to meet the needs of people viewing the scenery from the bridge (Figure 13(4-4)).

### 3.3. Effectiveness Assessment

**Figure 14:**
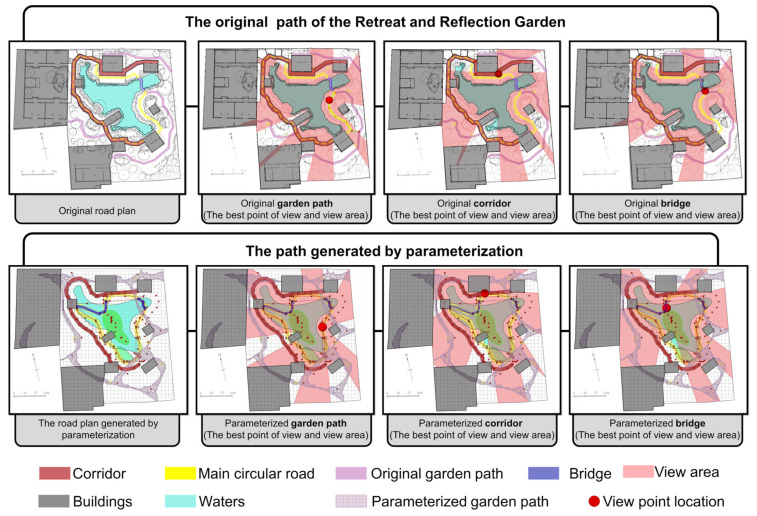
Comparison of original road and parametrically generated road in the Retreat and Reflection Garden.

After optimizing the generation process through the above parameterization and algorithm, the generated road can be compared with the original road for evaluation. Taking the Retreat and Reflection Garden as an example, it can be seen intuitively from Figure 14 that the layout of the newly generated road is very similar to the layout of the original path. At the same time, the calculation results of the best viewpoints for the three types of roads, namely, the garden path, corridor, and bridge, are also very close, especially as the best viewpoints of the garden path and corridor are almost the same. The newly generated bridge has an additional viewpoint compared to the original, and the result of the viewshed calculation is that the new bridge has a higher viewshed degree (Figure 14).

**Figure 15:**
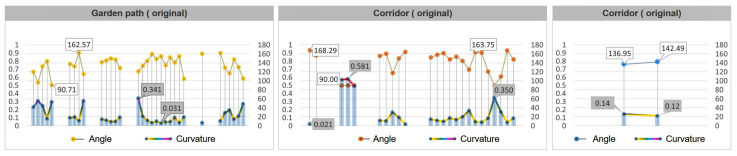
The original path and its angle and curvature.

**Figure 16:**
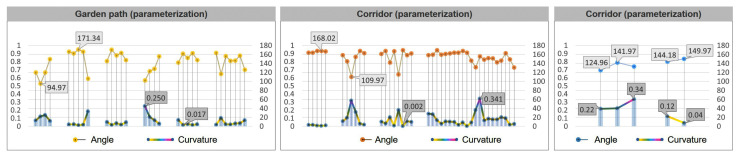
The path generated by parameterization and its angle and curvature.

From the data chart, it can also be seen that the effect of the parameterization and optimization algorithms not only continues the fluctuation trend and characteristics of the curvature and turning angle of the original path, but also increases the viewing area during the path-walking process. Comparing the curvature and angle values of the original road and the parametrically generated road, it was found that the angle value range of the original garden path was 90.71~162.57°. The parameterized garden path angle interval value is 94.97~171.34°. The original corridor path angle range was 90.00~168.29°, and the parameterized corridor angle range is 109.97~168.02°. The original bridge angle range was 136.95~142.49°. There are two bridges generated by parameterization, and the angle range values are 124.96~141.97° and 144.18~149.97°. Similarly, the road curvature of the parametrically generated road is very similar to the original path value (Figure 15 and Figure 16).

**Figure 17:**
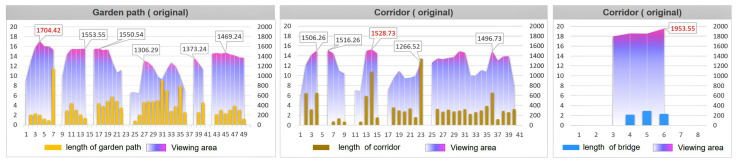
The superimposed comparison diagram of the length of one side of the road and the viewing area of the original garden path.

**Figure 18:**
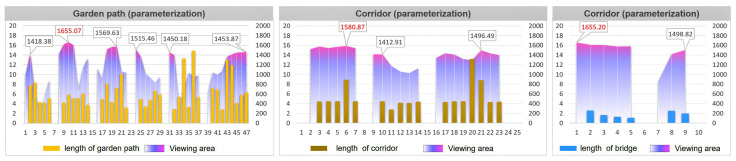
The comparison chart of the superposition of the length of one side of the road and the area of the viewshed generated by parameterization.

Evaluated from the viewshed aspect, the parametrically generated road has a larger viewshed visible area than the original road. The maximum areas of the original three types of roads were: garden path, 1704.42 m^2^; corridor, 1528.73 m^2^; and bridge, 1953.55 m^2^ (Figure 17). The maximum areas of the three types of road views generated by parameterization are: garden path, 1655.07 m^2^; corridor, 1580.87 m^2^; and bridge, 1655.20 m^2^ (Figure 18). After comparing the floor plan with the data, the research was shown to have obtained relatively intuitive and rational evaluation results. The results from the road curvature, angle, and field of view are obvious, which shows that the method of this study is feasible.

## 4. Discussion and Results: Application Effectiveness

In order to reflect the feasibility and application value of this research in modern applications, this research effort selected three practical projects and further applied the evaluated parameterized generation logic and optimization algorithm to them. They include 3000~10,000 square meters of courtyard green space (equivalent to the area of the Retreat and Reflection Garden), 10,000~20,000 square meters of community parks (equivalent to the area of the Canglang Pavilion), and 20,000–25,000 square meters of urban parks (equivalent to the area of the Humble Administrator’s Garden).

**Figure 19:**
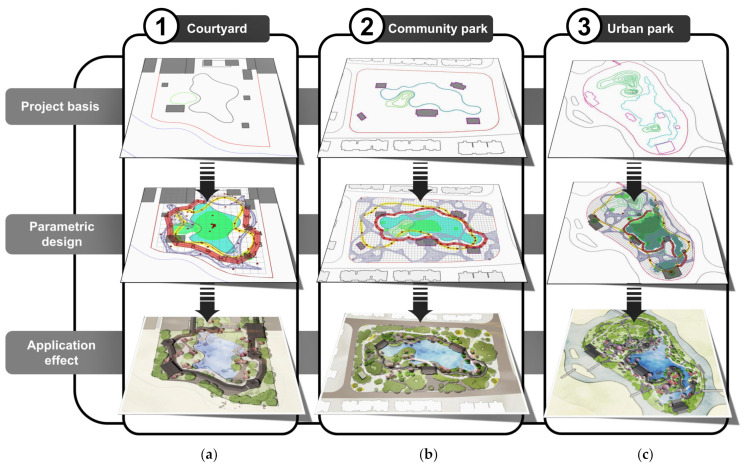
Parametric application effect: (**a**) application effect on courtyard green space; (**b**) application effect on community park; and (**c**) application effect on urban park.

Figure 19 shows the application effect of this study on courtyard green space (5307.29 m^2^), a community park (16,859.58 m^2^), and an urban park (22,598.35 m^2^). The site status was imported into Rhino, including red lines, reserved buildings, reserved landscapes, and water. According to the above parameterization generation logic and optimization process, the road system with the characteristics of classical gardens can be automatically generated (Figure 19).

**Figure 20:**
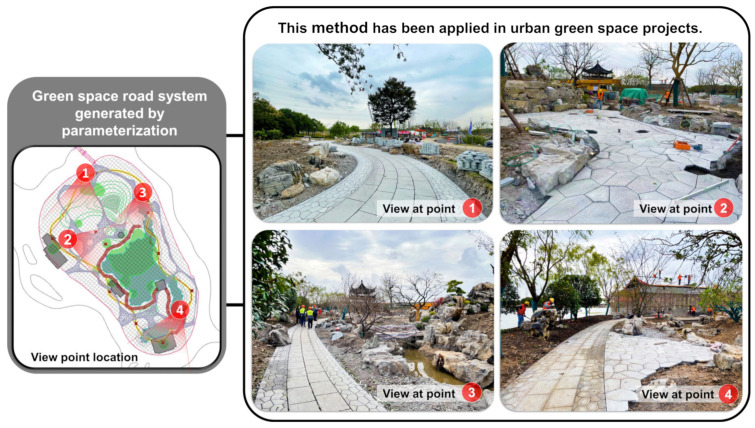
Application of intelligent road generation in urban green space. (Image source: photographed and annotated by the author.)

From the perspective of the application effect, it was successful. The road-system plan generated by the algorithm according to the current project inherited the characteristics of classical garden roads. Of the three applications, the road generation method obtained in this study has been applied to practical projects, and the construction of the urban park project in Figure 19c has started. The urban green space is part of Shanghai Pudong’s urban greening system. The park is located within Hengmian, Pudong, Shanghai. It is positioned as a park with Jiangnan garden characteristics, which will be completed and opened in early 2023. Therefore, our research results can be applied in practical projects. This not only verifies that the method of this study is feasible, but also that it has practical application value (Figure 20).

Nonetheless, our methods leave much to be desired. For example, not all the results obtained by the algorithm can be used directly. If the road is too complicated or broken, the designer needs to fine-tune it according to its actual situation. The actual uses of these findings face some limitations at present, and there are many areas to be improved in the future.

## 5. Conclusions

This paper took the garden road as a research object and proposed a method of generating paths for classical gardens based on parametric design. Through the practical application of research and design projects, the following conclusions were obtained:

(1) A parametric platform with Grasshopper as the core can effectively program the design of Jiangnan classical garden roads and reflect the relevant spatial characteristics through data input.
(2) After the programming of the Grasshopper parameterized platform has been completed, the global road network system can be further adjusted through the genetic algorithm to make the landscape more abundant.
(3) The slime mold algorithm has bionic characteristics, which has been reflected in the research of Jiangnan classical gardens, since ancient Chinese gardens have the characteristic of being integrated into the natural environment; however, if modern garden design utilizes the form of geometric cutting, this algorithm is not applicable.
(4) Judging from the effects of the projects constructed using landscape design practices that the researchers are developing at present, the method established in this study is effective.

The development of artificial intelligence has improved people’s efficiency and lessened the hardship of boring and repetitive work. However, it is difficult for computer programs to intelligently identify emotional factors in traditional culture. Chinese classical gardens carry the wisdom of the continuation of cultural thought, and modern design practitioners should consider the utilization of artificial intelligence from the perspective of sustainable design and the continuation of the historical context. This research not only identified the characteristics of landscape cultural heritage, but also developed an innovative, intelligent design tool. Urban green space roads are a part of urban systems. We hope that this intelligent generation method can be applied to more urban landscape spaces in the future.

## Data Availability

The datasets used and analyzed during the current study are available from the corresponding author on reasonable request.

## Appendix A

Grasshopper environment configuration: The operating system is Windows 11 (X64), the Cuda version is 11.5, the deep learning framework is Pytorch (1.13.0), the graphics card is GeForce GTX 3070 (16 G), and the processor is AMD Ryzen 9 5900HX (3.30 GHz).

## Appendix B

The table below renders the original data and statistics of the researchers on the road curvature, garden roads, corridors, and bridges of Jiangnan classical gardens. There are numbers on the corresponding floor plan in the statistical table, which represent the corresponding position of the data statistics.

**Table A1:** Summary of nine garden roads.

Name of Park		Road Type	Curvature (Min)	Curvature (Max)	Average (m^2^)	Angle (Min, °)	Angle (Max, °)	Average (m^2^)	Viewshed Area (Min, m^2^)	Viewshed Area (Max, m^2^)	Average (m^2^)
1	Humble Administrator’s Garden	Garden path	0.001208	0.636829	0.319018	106.204664	179.999978	143.102321	176.364453	8861.486339	4518.925396
		Corridor	0.004524	0.524093	0.264309	89.692108	177.879203	133.785656	1295.962864	6298.226974	3797.094919
		Bridge	0.031051	0.367986	0.199518	118.965893	175.103352	147.034623	1841.560527	9082.299147	5461.929837
2	Lingering garden	Garden path	1.26 × 10^−8^	0.617561	0.308781	88.832026	179.999995	134.416011	633.696715	4350.434777	2492.065746
		Corridor	0.000253	0.461852	0.231053	88.119789	179.999999	134.059894	15.031317	3279.972007	1647.501662
		Bridge	0.134161	0.296387	0.215274	138.777677	161.537099	150.157388	2129.081722	2240.850596	2184.966159
3	Canglang Pavilion	Garden path	0.008645	0.707684	0.358164	72.130602	174.769650	123.450126	20.383077	4272.086137	2146.234607
		Corridor	0.001115	0.316718	0.158916	88.919387	179.933871	134.426629	99.419211	2447.019617	1273.219414
		Bridge	0.058799	0.097450	0.078125	158.698048	166.278582	162.488315	2309.824214	2856.891253	2583.357734
4	Master of the Nets Garden	Garden path	0.004591	0.525293	0.264942	101.851392	178.184345	140.017869	67.712073	1377.772354	722.742214
		Corridor	0.000154	0.547225	0.273689	89.272999	179.933871	134.603435	95.123125	1376.995592	736.059359
		Bridge	0.164859	0.306599	0.235729	158.480635	166.278582	162.379609	909.809115	1241.671303	1075.740209
5	Garden of Harmony	Garden path	0.019605	0.672653	0.346129	77.091521	174.906341	125.998931	91.195824	2338.159116	1214.677470
		Corridor	0.013665	0.648475	0.331070	75.369205	175.885103	125.627154	18.555555	1770.190700	894.373128
		Bridge	0.246696	0.361807	0.304251	139.337049	147.935408	143.636229	1397.218074	1875.636620	1636.427347
6	Lion Forest Garden	Garden path	0.044481	0.709506	0.376993	83.112447	171.004655	127.058551	132.852701	2844.405519	1488.629110
		Corridor	0.025345	0.622304	0.323825	86.223376	175.211816	130.717596	30.669805	2347.352560	1189.011183
		Bridge	0.076988	0.406812	0.241900	126.107807	171.469741	148.788774	2140.667650	2396.741293	2268.704472
7	Retreat and Reflection Garde	Garden path	0.031021	0.539413	0.285217	82.905634	165.310415	124.108025	636.952331	1704.418149	1170.685240
		Corridor	0.018116	0.643158	0.330637	87.878286	168.292461	128.085374	403.003530	1528.728816	965.866173
		Bridge	0.122812	0.144795	0.133803	136.948404	142.490057	139.719231	1796.019980	1953.548509	1874.784245
8	Couple’s Garden Retreat	Garden path	0.016416	0.616795	0.316605	58.362688	178.578245	118.470467	103.250000	1636.882685	870.066343
		Corridor	0.017622	0.601354	0.309488	86.768515	175.472183	131.120349	62.210000	1101.042324	581.626162
		Bridge	0.336915	0.344940	0.340927	127.290767	129.904600	128.597684	1078.271105	1144.077125	1111.174115
9	Jichang Garden	Garden path	0.000007	0.662689	0.331348	88.622848	179.999967	134.311408	209.985126	3896.201775	2053.093451
		Corridor	0.129417	0.406271	0.267844	82.085717	163.676072	122.880895	8.221111	3644.391825	1826.306468
		Bridge	0.011054	0.011054	0.011054	154.791331	154.791331	154.791331	2682.586158	3545.397410	3113.991784
The comprehensive results of nine garden roads		Garden path	1.263 × 10^−8^	0.806	0.323	58.363	180.000	130.104	20.383	8861.486	1853.013
		Corridor	1.538 × 10^−4^	0.648	0.277	75.369	180.000	130.590	8.221	6298.227	1434.562
		Bridge	1.105 × 10^−2^	0.407	0.196	118.966	175.103	148.621	909.809	9082.299	2367.897

**Table A2:** Humble Administrator’s Garden, garden path.

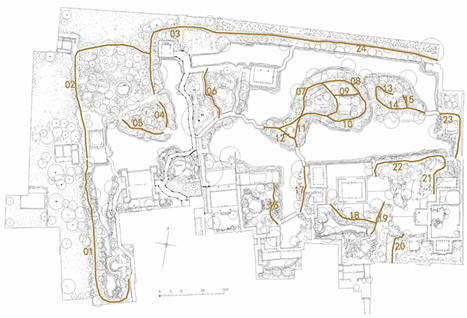

**Road No.**	**Angle No.**	**Curvature**	**Value of Angle (°)**	**Unilateral Length of Path (m)**	**Viewshed Area (m^2^)**
01	0	/	/	/	4063.743944
	1	0.084000396	162.954566	3.299322	4312.091742
	2	0.131870983	153.434391	3.757532	4494.553092
	3	0.278845938	140.38569	3.210439	3998.242655
	4	0.358836033	133.570789	1.616364	3917.547323
	5	0.04963161	169.792295	2.750569	3243.825878
	6	0.065208324	164.953042	4.417614	3022.884373
	7	0.108900494	158.825672	3.613321	3139.236447
	8	0.053884581	169.603479	3.134673	3118.296496
	9	0.019088623	170.957482	3.590857	3003.830228
	10	0.002495253	178.254303	12.911217	2759.244701
	11	0.016776015	172.1315	11.508717	2747.441927
	12	0.034052335	171.832217	4.844325	2720.261432
	13	0.113226513	160.763139	3.520742	2764.489166
	14	0.182507595	149.060086	2.3788	2696.490027
	15	0.102039514	155.821364	3.4595	2420.342468
	16	0.109624484	153.718431	4.745886	1691.894834
	17	0.199925817	134.393145	3.54477	1547.375737
	18	0.07307948	161.888424	4.204556	1430.076755
	19	/	/	4.410444	1414.203748
				Total length = 84.919646	
02	20	/	/	/	3615.307049
	21	0.072599593	162.204002	3.728924	3890.247338
	22	0.068908968	160.338936	4.791588	3796.068749
	23	0.031466376	169.033323	5.118985	3997.621154
	24	0.018991524	170.496017	6.02672	4138.725867
	25	0.013674969	174.40739	7.419394	4285.034976
	26	0.242869737	134.905379	3.846991	4367.867625
	27	0.111553938	151.806491	2.441236	4626.722292
	28	0.008242691	177.03933	6.239868	2497.772394
	29	0.009276512	176.821247	6.29674	2078.705406
	30	0.034688453	168.889695	5.663029	2098.748589
	31	0.017556467	174.545427	5.499628	2588.635346
	32	0.018727408	174.526041	5.341333	2535.706777
	33	0.049637142	165.691557	4.857851	2454.889076
	34	0.01016424	176.591231	5.178142	2540.540569
	35	0.013667244	176.591231	6.526666	3947.92151
	36	/	/	2.177211	4063.743944
				Total length = 85.154308	
03	37	/	/	/	5934.189428
	38	0.047725902	165.93313	5.433463	6076.700093
	39	0.01660293	174.713204	4.828913	5355.022474
	40	0.020966662	170.880648	6.282075	6341.559579
	41	0.031565464	168.552457	7.882949	4180.883712
	42	0.247594976	133.440614	3.744748	3791.128261
	43	0.080243306	161.681194	2.621894	3217.845359
	44	0.012392456	174.888564	5.301283	2501.084283
	45	0.013177369	174.864917	7.090662	5637.040375
	46	0.026867381	171.340026	4.505947	5287.53742
	47	/	/	6.733287	4578.426093
				Total length = 54.425221	
04	48	/	/	/	4578.426093
	49	0.111828404	158.659951	3.320472	4404.881246
	50	0.067117401	167.143853	3.302259	4397.808621
	51	0.107486568	162.334664	3.36998	4356.56113
	52	/	/	2.341965	4826.899637
				Total length = 12.334676	
05	53	/	/	/	1783.588619
	54	0.170397081	158.303002	2.798121	2581.323343
	55	0.119297813	175.118153	3.511082	2911.81879
	56	0.02704316	159.73107	2.788309	2221.18721
	57	0.119360007	179.999978	3.108156	2350.580055
	58	0.297378784	136.151384	2.020396	2584.087659
	59	0.066757774	169.726313	2.98682	2769.539394
	60	/	/	2.377664	2700.440561
				Total length= 19.590548	
06	61	/	/	/	5779.273848
	62	0.175627138	144.372225	4.106764	5566.709208
	63	0.046636701	169.241504	2.849138	5450.355322
	64	0.29369519	106.204664	5.18854	5627.457539
	65	0.259473113	124.689756	2.787275	5421.758525
	66	0.122687587	154.381776	4.322595	5437.437218
	67	0.248583991	130.387839	2.898382	5743.01255
	68	/	/	3.838629	5918.780829
				Total length = 25.991324	
07	69	/	/	/	8491.125317
	70	0.032090334	173.741861	3.504161	8401.262715
	71	0.09446084	152.674907	3.299802	7686.926987
	72	0.051892113	162.232504	4.668773	7425.590777
	73	0.111607887	150.038916	5.233049	8000.558688
	74	0.094790695	156.101518	3.025571	7971.986896
	75	0.091336425	155.979883	4.710163	8861.486339
	76	0.170570313	136.897933	4.402431	8156.596382
	77	0.066096561	167.272781	4.211396	7994.750242
	78	0.296241256	142.269799	2.493497	8168.254891
	79	0.101111598	159.611719	1.867167	8238.097244
	80	0.025697917	173.691574	5.110049	7902.627466
	81	0.145384413	151.282101	3.454141	6618.979084
	82	0.121259568	159.500775	3.369001	8142.664977
	83	0.294960422	121.338079	2.498506	8336.576569
	84	0.233557159	146.535895	4.08443	8484.642157
	85	/	/	0.742787	8382.629068
				Total length = 56.674924	
08	86	/	/	/	7980.472351
	87	0.030393644	173.158648	3.795863	7978.415785
	88	0.040163721	171.33327	4.056631	8109.117741
	89	0.116244958	157.889914	3.468381	7891.816325
	90	0.087530135	159.5575	3.129485	7896.688003
	91	0.087236111	161.774186	4.972902	6573.727799
	92	0.038145278	176.686525	2.276357	6104.139687
	93	/	/	0.755039	5839.945351
				Total length = 22.454659	
09	94	/	/	/	6095.743196
	95	0.048704067	169.533568	2.944208	5755.771645
	96	0.011627706	176.902506	4.545305	4346.887073
	97	/	/	4.752296	7499.178305
				Total length = 12.241808	
10	98	/	/	/	7951.226691
	99	0.107975508	159.834102	3.065746	7648.41234
	100	0.054665501	164.805102	3.419632	7624.697193
	101	0.064785495	161.402264	5.247287	7266.633246
	102	0.135986242	156.014977	3.720709	6595.190598
	103	0.158199972	155.917193	2.384607	6624.918475
	104	0.087951334	165.724805	2.889097	6581.665373
	105	0.081606369	164.298434	2.761849	6427.721526
	106	0.10182058	158.573908	3.931452	6167.006069
	107	0.221037974	148.163078	3.370444	6237.346148
	108	0.163774253	158.96458	1.566098	5645.680978
	109	0.217902056	142.284751	2.885485	5490.965533
	110	/	/	3.047521	5839.945351
				Total length = 39.289927	
11	111	/	/	/	7951.226691
	112	0.079129519	163.140676	3.838144	7687.163907
	113	0.018865062	176.311719	3.572148	5159.060784
	114	0.132954364	152.694869	3.25123	7228.60709
	115	/	/	3.848486	7816.411375
				Total length = 14.510009	
12	116	/	/	/	7592.449183
	117	0.019704269	176.281511	2.878293	6593.085835
	118	0.107288536	161.511999	3.707877	7988.722371
	119	/	/	2.276644	8228.60709
				Total length = 8.862813	
13	120	/	/	/	6321.750442
	121	0.039465708	174.515231	2.671991	6738.624443
	122	0.306088233	153.104284	2.17727	6889.809605
	123	0.636828682	119.970045	0.84512	6995.177447
	124	0.082086409	139.061251	1.09721	7009.26027
	125	0.16023737	171.941253	1.099338	6802.817194
	126	0.080108005	158.337582	2.323696	6697.387522
	127	/	/	2.367249	6483.988509
				Total length = 12.581874	
14	128	/	/	/	6321.750442
	129	0.054816352	168.630013	3.847622	6905.712761
	130	0.12891935	155.773958	3.380667	6881.596274
	131	0.040515982	172.383996	3.129871	6804.844976
	132	0.051760163	172.517084	3.42685	6615.794204
	133	0.117791832	168.334636	1.61459	6489.512893
	134	0.082425013	166.342238	1.836297	6040.286284
	135	0.021176456	175.810675	3.928576	6356.707367
	136	/	/	2.975348	6124.714272
				Total length = 24.13982	
15	137	/	/	/	2295.956837
	138	0.011577474	178.547975	3.010313	4677.812024
	139	0.228788913	159.507519	1.367478	5661.637963
	140	/	/	1.741707	5989.512893
				Total length = 6.119498	
16	141	/	/	/	1098.828483
	142	0.073544023	167.633613	3.587086	1527.677148
	143	0.156571239	159.270668	2.269311	1607.164429
	144	0.261061755	148.950591	2.32699	1651.105115
	145	0.227833237	160.497316	1.771106	1637.216939
	146	0.257654675	153.869873	1.200911	1655.85103
	147	0.18231048	154.326609	2.299288	1635.741497
	148	0.171311668	145.919097	2.574915	1594.32417
	149	/	/	4.248262	1556.834522
				Total length = 20.277869	
17	150	/	/	/	4124.476998
	151	0.019430875	175.050169	4.310572	3607.060663
	152	0.124065667	153.991218	4.57877	3785.558454
	153	0.06383749	166.66673	2.662776	2683.711052
	154	0.033242686	170.977824	4.607918	2278.173148
	155	/	/	4.856037	1922.96669
				Total length = 21.016074	
18	156	/	/	/	2639.912137
	157	0.162110865	144.566318	5.6306	2443.739851
	158	0.048214945	166.121711	2.615091	2525.844375
	159	0.057117471	161.668027	4.859369	2077.967228
	160	/	/	5.163593	1509.503889
				Total length = 18.268653	
19	161	/	/	/	1343.086678
	162	0.093803679	163.1153377	5.584201	1524.522148
	163	0.047118633	171.5186461	3.069767	1637.716474
	164	0.158195943	151.5247303	2.069169	1639.461889
	165	/	/	2.889481	1743.369426
				Total length = 13.612618	
20	166	/	/	/	395.704142
	167	0.322876973	131.735896	1.411931	384.385288
	168	0.239046266	116.113117	3.560727	374.969823
	169	0.031806694	169.953133	5.231021	444.496982
	170	/	/	5.65254	402.256083
				Total length = 15.856219	
21	171	/	/	/	1869.823947
	172	0.316887087	142.610377	2.617089	1001.974549
	173	0.252617287	146.36892	1.408122	847.730825
	174	0.18496448	150.384914	3.142499	511.376528
	175	0.225236479	138.594383	2.380792	301.594767
	176	0.01300723	176.827215	3.872076	176.364453
	177	/	/	4.641386	224.63895
				Total length = 18.061964	
22	178	/	/	/	2069.823947
	179	0.401474614	136.370625	1.919305	2280.671351
	180	0.097100415	161.275904	1.782701	2254.593069
	181	0.053103621	165.554558	2.898784	2072.530319
	182	0.059357615	165.80267	4.571421	2147.976028
	183	0.087305451	163.346445	3.755691	2003.225504
	184	0.048216263	165.85577	2.87809	2056.081979
	185	0.010089974	177.21431	6.320878	2188.61576
	186	0.072809464	167.611751	2.314583	2162.289797
	187	0.271212883	124.930439	3.611406	2234.808666
	188	0.212384385	144.98768	3.203342	2212.593829
	189	/	/	2.457123	1249.523985
				Total length = 35.713324	
23	190	/	/	/	2200.811309
	191	0.030348039	174.312192	3.484053	2183.306236
	192	0.084747473	166.878851	3.055403	2010.882532
	193	0.445173067	128.966176	2.336619	1455.323038
	194	0.090130447	157.255862	1.514066	923.807925
	195	0.027868932	171.061815	6.162724	1565.230157
	196	0.013417238	177.125663	4.018563	1986.316581
	197	0.052695276	169.591823	3.458621	1943.796366
	198	/	/	3.426522	2060.4625
				Total length = 27.456571	
24	199	/	/	/	5251.571586
	200	0.004166304	174.228716	20.751681	5090.443563
	201	0.020380382	165.31563	22.58122	4519.395705
	202	0.073133883	169.927597	2.365078	4574.633392
	203	0.033671194	174.187119	2.436255	4577.937156
	204	0.069074243	168.676857	3.587018	4645.29687
	205	0.060217888	168.922977	2.123962	4753.415413
	206	0.019009516	175.41104	4.283656	4623.44976
	207	0.009392593	176.625992	4.140652	4518.987793
	208	0.003353393	178.280642	8.396034	4160.550544
	209	0.001208208	179.378388	9.500703	3956.92425
	210	0.013118558	172.689991	8.45844	3071.860237
	211	/	/	10.978588	1946.835324
				Total length = 99.603287	
Maximum value		0.636828682	179.999978	22.58122	8861.486339
Minimum value		0.001208208	106.204664	0.742787	176.364453

**Table A3:** Lingering Garden, garden path.

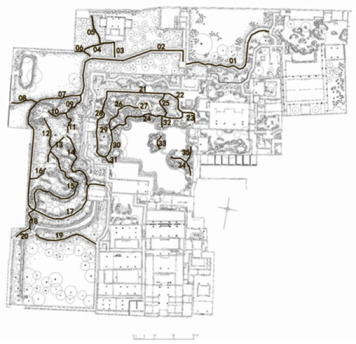

**Road No.**	**Angle No.**	**Curvatur**	**Value of Angle (°)**	**Unilateral Length of Path (m)**	**Viewshed Area (m^2^)**
01	0	/	/	/	3535.82797
	1	0.06390093	167.950905	4.978056	2681.138077
	2	0.292068665	152.991576	1.581987	2605.098089
	3	0.116985781	169.062109	1.616114	2895.971742
	4	0.351103506	149.746186	1.642624	2849.075513
	5	0.23749044	156.410039	1.329169	2792.801511
	6	0.082194573	170.209895	2.109808	2851.13719
	7	0.246860721	148.71703	2.042825	2665.55825
	8	0.161300121	151.553287	2.325196	2800.861418
	9	0.163364367	148.408235	3.75704	2599.076868
	10	0.238892428	135.196854	2.903711	2456.556953
	11	0.284531428	134.882971	3.473015	2313.821396
	12	0.324907102	142.373166	1.879297	2335.662872
	13	0.198624361	150.682805	2.090264	2422.079703
	14	0.011825784	177.571557	3.000406	2314.271217
	15	0.006312791	178.549569	4.167088	2366.096022
	16	0.082551496	161.169271	3.852894	2372.890984
	17	0.153552463	143.532921	4.07373	2400.652496
	18	0.104490119	159.520742	4.076995	2447.812097
	19	0.151299988	157.844094	2.723674	2440.776025
	20	/	/	2.355639	2456.393097
				Total length = 54.650363	
02	21	/	/	/	1928.712504
	22	0.119474243	88.832026	5.517773	1687.972166
	23	0.022439619	160.158172	15.913685	1904.084522
	24	0.027467031	156.222078	14.797205	2065.731987
	25	0.040717425	159.568422	15.204552	3577.213193
	26	0.148630391	162.308209	2.056683	4350.434777
	27	0.175833896	133.67384	2.081836	4277.314366
	28	0.045144951	164.633802	6.650293	3432.10358
	29	0.072769076	161.645105	5.193825	3496.906086
	30	/	/	3.569304	3509.746218
				Total length = 70.985156	
03	31	/	/	/	2306.383349
	32	0.026334549	174.801649	0.451703	2261.305108
	33	0.010523033	177.863173	6.43104	2037.719048
	34	/	/	0.655786	1989.08065
				Total length = 7.53853	
04	35	/	/	/	1989.08065
	36	0.050624113	172.577176	1.512429	2055.695162
	37	0.073051464	166.974265	3.600417	2484.24283
	38	0.027012946	175.385022	2.609307	2636.153119
	39	0.049267349	169.83512	3.352553	3899.580251
	40	0.243930492	142.090311	3.839838	3778.867776
	41	/	/	1.421488	4235.865002
				Total length = 16.336032	
05	42	/	/	/	2308.537615
	43	0.073309583	169.760573	1.203013	2622.016476
	44	0.032943738	179.999987	2.903014	2628.043908
	45	0.147444414	164.739111	1.267354	2584.089311
	46	0.059024965	168.005601	1.267354	2728.866968
	47	0.104316786	178.824229	1.491424	2587.733918
	48	0.137348758	160.448135	2.428814	2175.646333
	49	4.441 × 10^−8^	168.852755	2.183073	2418.05731
	50	/	/	2.366415	2695.600512
				Total length = 15.110462	
06	51	/	/	/	4208.770587
	52	0.466845548	160.040377	0.830431	4082.225005
	53	0.258974893	161.951306	1.589264	4231.391381
	54	0.26106333	160.394807	1.017488	4162.526998
	55	0.003488635	135.182685	0.749726	4235.865002
	56	/	/	3.82218	4004.06058
				Total length = 8.009089	
07	57	/	/		3509.746218
	58	0.432195186	139.570592	1.510894	3505.502331
	59	0.073777348	170.796431	1.686442	3305.9875
	60	0.058985184	169.858306	2.662689	3175.410455
	61	0.056435308	170.936308	3.330894	3060.505491
	62	0.0979961	160.794614	2.268748	3077.729283
	63	0.021538069	175.582405	4.529555	3247.257113
	64	0.059251443	169.986778	2.627868	2468.884536
	65	0.134311691	159.882209	3.263406	2327.132649
	66	0.068135664	166.167845	1.93289	2960.057821
	67	0.104636085	151.702197	5.125635	3311.498414
	68	0.069424769	166.678565	4.216045	2322.64164
	69	0.340224385	134.523087	2.463724	2089.124622
	70	1.32421 × 10^−8^	179.999995	2.077753	2150.855958
	71	0.344107151	134.523082	1.768003	3314.939594
	72	0.011845401	174.950011	2.707782	3177.002491
	73	6.13275 × 10^−6^	179.994647	12.163109	3731.98411
	74	0.007716559	174.944658	18.292679	3876.608922
	75	/	/	4.560183	3751.984018
				Total length = 77.188297	
08	76	/	/	/	2960.057821
	77	0.192802253	147.635339	3.067503	3517.589119
	78	0.101839225	163.294847	2.713566	3040.981211
	79	0.102129408	166.421362	2.991924	2468.556228
	80	0.112349659	165.760732	1.635427	2478.288878
	80	/	/	2.774977	2221.226423
				Total length = 13.183396	
09	82	/	/	/	3505.502331
	83	0.416151619	134.337759	2.710609	3610.72118
	84	0.254942506	159.651721	0.944055	3485.359317
	85	0.273648243	151.44263	1.822904	3495.097051
	86	0.410541106	138.933691	1.782271	3439.548866
	87	0.178548684	151.676156	1.634716	3046.748049
	88	/	/	3.818658	2628.963551
				Total length = 12.713213	
10	89	/	/	/	1787.641237
	90	0.185666609	155.183579	2.128449	3736.731945
	91	0.247248333	155.455758	2.5001	3687.325984
	92	0.612855377	132.648587	0.921439	3582.214126
	93	0.367555998	138.985512	1.678372	3298.96002
	94	0.069743471	173.663075	2.130368	2632.333225
	95	/	/	1.03907	2628.963551
				Total length = 10.397798	
11	96				2628.963551
	97	0.198379619	166.093135	1.236458	2522.114935
	98	0.572707948	133.218776	1.20457	2477.98976
	99	0.602972309	119.794006	1.563845	3066.625674
	100	0.120086729	133.042411	1.716912	3210.813133
	101	0.178184178	168.508509	1.617813	2716.591909
	102	/	/	3.020261	2090.539594
				Total length = 10.359859	
12	103	/	/	/	3288.028501
	104	0.25916738	152.342112	1.398901	3108.21679
	105	0.205330344	152.607919	2.283769	3227.608144
	106	0.132719701	160.949625	2.328709	3415.584586
	107	0.186298593	150.511224	2.658373	3442.85236
	108	0.013469241	177.237639	2.805988	3339.836727
	109	/	/	4.352054	2473.506432
				Total length = 15.827794	
13	110	/	/		2943.942818
	111	0.129116363	154.29058	8.048674	3288.028501
	112	0.019517653	177.36145	1.73571	2936.456555
	113	0.1528815	159.232158	2.982751	2218.235698
	114	/	/	2.556772	1673.786865
				Total length = 15.323907	
14	115	/	/	/	2684.987796
	116	0.193026675	152.942695	1.940786	3674.761723
	117	0.012657299	177.580259	2.42697	3938.986463
	118	0.256173607	136.664715	4.245668	4090.899009
	119	0.29236638	124.003564	1.408229	4044.625267
	120	0.338288773	162.813658	1.261943	4077.191695
	121	0.124857659	115.022711	0.781012	4090.692179
	122	0.067739914	154.66406	1.8898	4099.180519
	123	0.479772688	165.900852	2.041941	4137.22177
	124	0.336394762	172.781247	1.675381	4230.273696
	125	0.518537436	137.334986	1.354968	4253.286289
	126	0.235131567	158.799375	2.864276	4284.335696
	127	0.334159145	119.614895	1.718253	4274.93081
	128	0.169825636	148.667155	1.822169	4197.201049
	129	0.064326012	145.591604	1.429054	3249.607924
	130	0.331456888	164.129351	1.68965	3191.319675
	131	0.536620151	174.250393	1.798687	3150.64823
	132	/	/	1.700754	3126.832445
				Total length = 33.568943	
15	133	/	/	/	1800.218045
	134	0.01961355	176.765985	3.012347	2545.013188
	135	0.193569632	150.857159	2.742543	3125.773871
	136	0.085958783	171.363418	2.455804	3266.913968
	137	0.309137584	159.309831	1.276736	3287.018784
	138	0.20502035	167.462423	1.156774	3312.080938
	139	0.440054684	107.325825	1.149718	3306.076958
	140	0.430381479	150.601411	2.409635	3252.074454
	141	0.433301011	134.441987	1.532142	3201.521056
	142	0.549113823	129.143674	1.15402	3244.626427
	143	0.316211988	136.651596	1.662707	3223.979247
	144	0.307389954	118.893352	2.001406	3142.396342
	145	0.090312004	156.40469	3.586578	3145.077891
	146	0.26218264	109.327289	2.225792	3241.008794
	147	0.155288564	104.329969	3.7935	3465.911399
	148	0.117703494	141.096902	3.265311	3448.373123
	149	0.076004585	164.911143	4.19934	3433.068609
	150	0.359191295	133.214111	2.854091	3427.894705
	151	0.280897946	148.18319	1.314139	3304.539003
	152	0.157091852	154.612324	1.873408	3105.969512
	153	0.448618904	164.590565	1.44457	3068.37175
	154	0.38326031	135.512653	1.83763	3214.635014
	155	0.245173367	154.108826	2.214497	3370.349029
	156	0.071843099	165.023188	3.236642	3366.357463
	157	0.202763784	136.750187	2.378183	3336.461322
	158	0.167729144	134.270992	1.925553	3321.24284
	159	/	/	1.015808	3272.837881
				Total length = 61.155483	
16	160	/	/	/	3112.944103
	161	0.220151654	142.758914	3.880997	3278.632641
	162	0.136060978	164.96833	1.881178	3321.239101
	163	0.587821874	119.305504	1.964144	3323.517878
	164	0.494672028	137.261323	1.461271	3310.972896
	165	/	/	1.4799	3144.762417
				Total length = 10.667491	
17	166	0.14628209	155.054042	2.182493	3276.167858
	167	0.005978625	178.464032	3.713611	3324.483313
	168	0.026489548	173.339913	5.253842	3362.48673
	169	0.090798375	160.154263	3.516955	3238.061891
	170	0.068922221	160.001449	4.073844	3095.145232
	171	0.053051398	163.142911	5.997646	3457.064835
	172	0.08010435	157.503907	5.053073	3228.833539
	173	0.086326787	153.104567	4.686798	3133.980204
	174	0.110840366	153.040081	6.083726	2774.090936
	175	0.389865768	120.712754	2.278983	2238.219027
	176	0.039738547	171.254406	2.787366	633.696715
	177	/	/	4.885681	761.262665
				Total length = 50.514019	
18	178	/	/	/	3319.189247
	179	1.26338 × 10^−8^	179.999959	1.499828	3305.528563
	180	0.617561312	135.329713	1.227516	3249.166569
	181	0.47612121	115.269643	1.233929	3254.872751
	182	0.548395992	113.353129	1.731739	3298.271139
	183	0.567854292	120.358101	2.827068	3309.715795
	184	0.342118891	133.387547	2.142903	3363.260535
	185	/	/	1.188963	3343.831703
				Total length = 12.411198	
19	186	/	/	/	3339.922244
	187	0.128334298	155.380662	2.771615	3306.283569
	188	0.05458412	167.433469	3.869071	3364.360455
	189	0.055987747	166.151058	4.151071	3267.204602
	190	0.051429745	166.354235	4.462217	3150.333653
	191	0.056159589	169.349947	4.777536	3261.980481
	192	0.228065725	148.935481	2.860884	3223.335
	193	/	/	7.857244	3287.73106
				Total length = 32.57651	3169.965416
20	194	/	/	/	3343.831703
	195	0.165670274	158.902625	2.5818	3285.207124
	196	0.175647774	150.488445	1.836143	2530.751013
	197	/	/	3.937616	2472.97785
				Total length = 8.355559	
21	198	/	/	/	2515.226609
	199	0.00034265	179.895586	5.397596	2403.284434
	200	0.000361598	179.75799	5.23904	2292.983804
	102	0.107800261	90.947686	18.123727	2683.370368
	202	/	/	3.666823	2622.946585
				Total length = 32.427185	
22	203	/	/	/	2515.226609
	204	0.139953515	149.605646	2.561886	2578.150461
	205	/	/	4.903313	2646.765425
				Total length = 7.4652	
23	206	/	/	/	2646.765425
	207	0.23478856	127.895364	4.829676	2646.669828
	208	0.172008666	144.451307	2.570715	2648.144483
	209	0.112577834	160.244628	4.501182	2494.079595
	210	0.348987734	115.040419	1.572621	2408.774939
	211	/	/	4.279165	1432.664177
				Total length = 17.753359	
24	212	/	/	/	2795.106121
	213	0.034886526	174.799724	3.037242	2763.751879
	214	0.396585773	130.016823	2.164081	2760.053556
	215	0.347552337	143.561077	2.09703	2732.787591
	216	0.419475336	98.641986	1.495925	2726.363611
	217	0.436073243	141.137962	1.754537	2714.931084
	218	0.340998033	150.669374	1.415472	2693.746255
	219	0.286105062	159.008973	0.902943	2678.334358
	220	0.072067128	151.085203	1.232952	2670.455481
	221	0.208207836	167.821294	2.247696	2697.03136
	222	0.200830246	147.118639	3.638231	2719.273884
	223	0.189416656	157.631999	1.771093	2685.36221
	224	0.188999677	147.83726	2.091564	2577.307263
	225	0.131315528	147.077628	3.738715	2613.481188
	226	0.054683167	152.166635	2.242133	2592.233801
	227	0.129367039	164.411183	5.050931	2626.047916
	228	0.580466103	156.832514	4.869379	2612.041766
	229	/	/	1.295958	2616.596778
				Total length = 43.471071	
25	230	/	/	/	2515.226609
	231	0.226627661	148.15504	2.683099	2563.575266
	232	0.568557691	109.415115	2.156641	2567.155346
	233	0.202679242	155.10268	1.904098	2601.892817
	234	0.097405042	169.261594	2.349068	2586.367122
	235	0.012549879	178.981848	1.492734	2605.561003
	236	0.425566386	108.506172	1.339075	2589.533842
	237	/	/	1.16168	2610.845824
				Total length = 13.086395	
26	238	/	/	/	2761.609952
	239	0.217802543	142.556716	2.648624	2710.057926
	240	0.228186313	150.077115	3.242646	2735.651035
	241	0.325621654	147.962774	1.251549	2677.357622
	242	0.599666776	109.197723	2.128899	2747.33552
	243	0.176457761	160.960993	1.724528	2687.315391
	244	0.254041084	145.796875	2.024092	2646.833414
	245	0.217009254	148.743351	2.602701	2571.013966
	246	0.213094836	152.776955	2.362456	2466.406159
	247	/	/	2.054432	2285.272496
				Total length = 20.039926	
27	248	/	/	/	2285.272496
	249	0.478035155	130.831472	1.774131	2271.066032
	250	0.307976788	137.999694	1.706908	1743.093288
	251	0.359139193	120.000035	2.924091	1911.479572
	252	0.384034908	120.029721	2.642402	2280.408787
	253	0.366031434	122.396607	2.562918	2481.930533
	254	0.346970734	128.846726	2.701428	2532.761737
	255	/	/	2.271309	2285.272496
				Total length = 16.583187	
28	256	/	/	/	2622.946585
	257	0.384570366	150.151126	1.814076	2626.143011
	258	0.170104127	163.48226	0.85241	2656.162005
	259	0.089726239	170.007308	2.516767	2635.275363
	260	0.428865567	137.579032	1.36449	2645.492777
	261	0.535215059	128.028077	2.000899	2674.379915
	262	0.339579735	136.685373	1.25334	2658.754178
	263	0.316964248	138.459868	3.035845	2588.971406
	264	0.197288471	158.931653	1.395909	2619.199957
	265	/	/	2.306912	2627.553473
				Total length = 16.540649	
29	266	/	/	/	2761.609952
	267	0.0718809	169.614711	2.0923	2732.438262
	268	0.308329422	143.0707	2.943476	2684.581315
	269	/	/	1.120006	2627.553473
				Total length = 6.155783	
30	270	/	/	/	2627.553473
	271	0.1008721	162.328489	3.10199	2667.381925
	272	0.026919388	174.708536	2.988965	2669.393697
	273	0.066967472	167.283788	3.869973	2667.452095
	274	0.375668574	127.6362	2.743515	2647.628611
	275	0.196886852	156.602864	1.937745	2675.434918
	276	0.333122694	139.940744	2.181326	2717.481469
	277	0.338140055	138.796207	1.930328	2789.544386
	278	0.072748008	171.770338	2.23059	2838.671691
	279	0.270987148	155.995515	1.714671	2805.022269
	280	0.138007706	163.531893	1.353888	2843.85603
	281	0.163849899	154.680984	2.791882	2810.563992
	282	0.04412452	174.481731	2.558041	2788.819346
	283	0.252076525	155.965592	1.805574	2800.709584
	284	0.205166821	159.968367	1.497621	2790.118361
	285	0.320496068	154.575017	1.892469	2772.84921
	286	/	/	0.843807	2761.609952
				Total length = 35.442384	
31	287	/	/	/	2734.142435
	288	0.309763837	126.958402	3.069075	2802.292772
	289	/	/	2.69386	2749.722818
				Total length = 5.762935	
32	290	/	/	/	2716.794823
	291	0.073019676	172.94046	1.78252	2675.833284
	292	0.069767169	173.652583	1.5901	2642.013492
	293	0.142754268	170.758606	1.584089	2609.874735
	294	/	/	0.671979	2590.511495
				Total length = 5.628689	
33	295	/	/	/	2766.310978
	296	0.244401082	156.944292	1.696309	2774.585408
	297	0.565341808	128.17651	1.574356	2783.899224
	298	0.215016579	152.372891	1.517357	2790.683228
	299	/	/	2.911166	2781.948192
				Total length = 7.699188	
34	300	/	/	/	2737.313852
	301	0.212866597	148.121048	1.509983	2553.940714
	302	0.174121536	145.158168	3.615317	1036.247006
	303	0.112594716	161.414268	3.261492	1565.684101
	304	0.146321901	156.465561	2.473777	1800.070455
	305	0.094367258	166.962003	3.099693	2003.358006
	306	/	/	1.710061	2134.823488
				Total length = 15.670322	
35	307	/	/	/	1565.684101
	308	0.116897247	161.68685	2.896027	1703.088035
	309	0.10100498	163.907109	2.548906	1972.778605
	310	/	/	2.994061	2230.13591
				Total length = 8.438995	
Maximum value		0.617561312	179.999995	18.123727	4350.434777
Minimum value		1.26338 × 10^−8^	88.832026	0.671979	633.696715

**Table A4:** Canglang Pavilion, garden path.

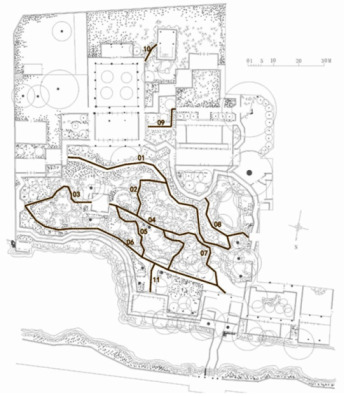

**Road No**.	**Angle No.**	**Curvature**	**Value of Angle (°)**	**Unilateral Length of Path (m)**	**Viewshed Area (m^2^)**
01	0	/	/	/	1506.307158
	1	0.03818748	161.824278	4.498802	1810.796905
	2	0.063337486	144.31005	12.002179	1931.26802
	3	0.019018214	172.126657	7.29131	1908.01948
	4	0.065768033	158.523872	7.148304	2061.185869
	5	0.101764716	146.525281	4.169509	2130.369115
	6	0.060710436	155.826627	7.115414	2071.094767
	7	0.089380305	150.916204	6.680341	1905.882194
	8	0.113944545	147.035878	4.542802	1764.971815
	9	0.097176786	158.467248	5.413631	1805.830762
	10	0.139566821	128.481061	2.252117	1848.537189
	11	0.058663963	156.990536	9.676967	706.344901
	12	0.173795277	132.669136	3.871007	693.456081
	13	0.029052408	171.845291	5.344736	1950.536885
	14	0.355151426	116.012167	4.444703	2185.122634
	15	0.345378205	116.521951	1.158365	2248.019691
	16	/	/	4.559376	2338.837581
				Total length = 90.169562	
02	17	/	/	/	4026.118458
	18	0.030207494	167.728295	6.896737	4035.517852
	19	0.480696334	72.130602	4.32605	3980.217252
	20	0.10963525	140.88756	1.925977	3979.432779
	21	0.171493207	142.345592	3.694784	3927.154933
	22	0.147154672	142.57582	3.832251	3909.093917
	23	/	/	4.880936	4076.123226
				Total length = 25.556734	
03	24	/	/	/	2439.011224
	25	0.086668885	143.325764	11.346066	2513.11784
	26	0.105247983	160.557134	2.901612	2460.824128
	27	0.271726145	138.489619	3.51506	2432.418384
	28	0.403469506	119.930778	1.653689	2395.670275
	29	0.161313675	157.351432	3.224668	2092.612213
	30	0.409906969	137.378141	1.633967	1996.031701
	31	0.180796705	132.621859	1.91084	1954.749645
	32	/	/	6.343989	999.492895
				Total length = 32.52989	
04	33	/	/	/	3230.605
	34	0.033403617	168.65042	2.789863	3455.006771
	35	0.017956399	172.125395	9.034741	4076.123226
	36	0.031171655	169.318725	6.259966	4162.133213
	37	0.199743713	129.054718	5.683692	4213.489877
	38	0.279130937	133.091452	2.820233	4255.54116
	39	0.128893908	158.849424	2.883364	4272.086137
	40	0.165923023	155.830842	2.812076	4251.631818
	41	0.707683651	103.785243	2.233453	4247.858051
	42	0.149169086	160.590286	1.149588	4238.59288
	43	0.111752261	161.785421	3.35496	4247.075546
	44	0.130903701	150.498165	2.30808	4215.151709
	44	/	/	5.42871	4107.055534
				Total length = 46.758727	
05	45	/	/	/	3455.006771
	46	0.173605554	141.169571	4.778766	3832.660103
	47	0.170186683	145.806034	2.850016	3986.720014
	48	0.359506542	112.396127	4.049944	4122.013562
	49	0.119854915	162.998765	1.983297	4133.106846
	50	0.302092911	137.284807	2.947898	4194.063909
	51	0.474221905	118.950501	1.85533	4197.304171
	52	0.241825554	140.478568	2.416052	4137.046151
	53	/	/	3.169731	4071.71557
				Total length = 26.922123	
06	54	/	/	/	2439.011224
	55	0.056033061	161.717867	7.294444	2399.058774
	56	0.072158783	160.157835	4.03428	2314.544719
	57	0.063280201	160.419638	5.512904	2486.34169
	58	0.011946445	174.625267	5.23542	2800.29349
	59	0.044393546	154.879396	10.461477	3596.515564
	60	0.022252245	166.699595	9.130754	3942.089914
	61	0.019504868	167.057573	11.684329	3940.523889
	62	0.008644812	172.097281	11.428644	4107.055534
	63	0.0343426	155.148803	20.450103	4041.223857
	64	/	/	4.359658	3763.238173
				Total length = 89.592013	
07	65	/	/	/	4213.489877
	66	0.02715409	174.76965	3.437047	4268.989415
	67	0.169037397	145.865338	3.284215	4257.324721
	68	0.153493878	151.170449	3.659926	4185.937413
	69	0.31149141	129.918911	2.823803	4177.051668
	70	0.181693273	154.509447	2.610545	4183.941799
	71	0.089480263	166.207756	2.245662	4128.287131
	72	0.371801851	122.657598	3.120719	4161.98264
	73	0.1311243	154.225436	2.004785	4159.525012
	74	0.240988308	111.142837	4.769477	4209.32205
	75	0.145704932	137.475827	4.614252	4139.734722
	76	/	/	5.33709	4041.223857
				Total length = 37.907522	
08	77	/	/	/	3870.524325
	78	0.226819196	133.366326	2.453076	3947.195156
	79	0.135305485	140.892538	4.47271	4028.035728
	80	0.089799877	147.890889	5.415989	4126.07339
	81	0.110937107	136.576624	6.895267	4136.49217
	82	0.187917267	127.596218	6.442106	3995.349025
	83	/	/	2.78222	3906.051075
				Total length = 28.461368	
09	84	/	/	/	779.408479
	85	0.171448413	92.296238	9.118068	718.541637
	86	0.280907054	92.296238	6.90485	628.299402
	87	/	/	1.458185	20.383077
				Total length = 17.481102	
10	88	/	/	/	250.125543
	89	0.088726513	159.252064	5.56931	431.611283
	90	/	/	2.529671	446.304538
				Total length = 8.098981	
11	91	/	/	/	1132.863781
	92	0.066680799	157.846181	8.373149	1218.838083
	93	/	/	3.105771	1223.566358
				Total length = 11.47892	
Maximum value		0.707683651	174.76965	20.450103	4272.086137
Minimum value		0.008644812	72.130602	1.149588	20.383077

**Table A5:** Master of the Nets Garden, garden path.

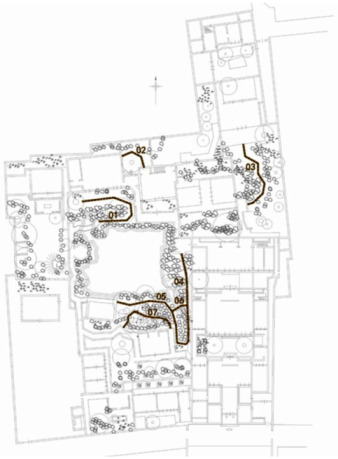

**Road No.**	**Angle No.**	**Curvature**	**Value of Angle (°)**	**Unilateral Length of Path (m)**	**Viewshed Area (m^2^)**
01	0	/	/	/	1241.671303
	1	0.144057347	134.50147	8.322068	1373.261141
	2	0.295304988	132.220285	2.092735	1377.772354
	3	0.242252077	116.484344	3.363873	1315.451558
	4	0.065275094	152.08979	5.248122	1238.76037
	5	/	/	9.492359	1143.859196
				Total length = 28.519157	
02	6	/	/	/	74.867076
	7	0.271683432	129.577471	3.185318	77.841276
	8	0.246368754	133.021186	3.085881	76.501852
	9	/	/	2.984779	67.712073
				Total length = 9.255978	
03	10	/	/	/	472.169964
	11	0.168883951	152.14342	1.679867	475.356937
	12	0.177340551	143.589531	3.992341	480.472682
	13	0.222383672	136.793335	3.047629	481.863439
	14	0.172536621	146.915893	3.571524	482.191033
	15	0.275131359	118.81849	3.027275	483.680254
	16	0.220778204	127.367189	4.331148	483.896734
	17	/	/	3.69478	484.114435
				Total length = 23.344562	
04	18	/	/	/	686.585881
	19	0.110008938	168.466448	0.237224	705.263839
	20	0.01732888	175.902949	3.402291	987.675646
	21	0.004591228	178.184345	4.848731	1161.777249
	22	0.011770549	176.112315	8.954698	1156.323219
	23	0.030463781	172.310389	2.570309	1182.397398
	24	/	/	6.23069	771.729126
				Total length = 26.243943	
05	25	/	/	/	1298.121331
	26	0.090685637	154.235996	3.910399	1343.873545
	27	0.057217492	162.705227	5.912635	1148.230565
	28	0.193975822	134.258794	4.596445	1098.99657
	29	0.103480318	157.015892	3.402133	945.305673
	30	0.045315087	167.54692	4.296968	659.305301
	31	0.306194793	101.851392	5.276281	346.498125
	32	0.525292881	117.444636	2.684656	562.681992
	33	/	/	1.158616	686.585881
				Total length = 31.238134	
06	34	/	/	/	945.305673
	35	0.206821803	149.851364	3.316176	1170.920396
	36	/	/	1.694702	1172.529369
				Total length = 5.010877	
07	37	/	/	/	1036.643308
	38	0.346124468	141.177208	2.417478	1182.676001
	39	0.463069662	126.612089	1.406778	1216.630905
	40	0.362483592	117.422288	2.438779	1305.408914
	41	0.076022842	167.443653	3.269966	781.406918
	42	0.096135568	168.477726	2.483218	362.673971
	43	0.116944925	166.507327	1.692681	298.442782
	44	0.341374979	139.83492	2.32472	323.894588
	45	0.284831852	154.487645	1.692041	424.607785
	46	/	/	1.408105	451.513499
				Total length = 19.133766	
Maximum value		0.525292881	178.184345	9.492359	1377.772354
Minimum value		0.004591228	101.851392	0.237224	67.712073

**Table A6:** Garden of Harmony, garden path.

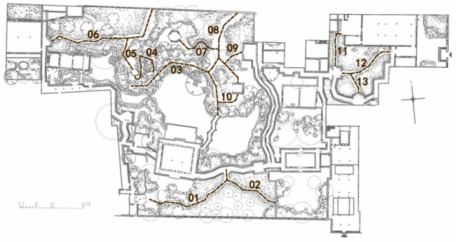

**Road No.**	**Angle No.**	**Curvature**	**Value of Angle (°)**	**Unilateral Length of Path (m)**	**Viewshed Area (m^2^)**
01	0	/	/	/	1604.802601
	1	0.19695515	127.285112	3.245636	1569.226596
	2	0.068150907	165.628111	5.689282	1415.071007
	3	0.297955222	148.450971	1.635256	1399.287988
	4	0.16439692	142.034725	2.013022	1366.436047
	5	0.1956945	132.327539	5.794324	1089.971381
	6	0.111236452	158.595738	2.322225	946.214613
	7	0.144101881	154.971405	4.344557	688.11648
	8	0.263836569	138.796349	1.640404	701.050855
	9	/	/	3.641104	676.938225
				Total length = 30.32581	
02	10	/	/	/	1569.226596
	11	0.122508283	160.105106	2.649015	1520.74656
	12	0.17290609	143.069305	2.990609	1489.213759
	13	0.13965808	142.765625	4.323146	1394.817797
	14	0.150751678	149.739073	4.818975	1130.560522
	15	/	/	2.066428	999.901286
				Total length = 16.848173	
03	16	/	/	/	1799.105567
	17	0.01960484	174.906341	4.068621	1790.453517
	18	0.079697761	157.395071	4.996746	1754.407379
	19	0.130547189	141.525379	4.840126	1676.722792
	20	0.041432046	166.054799	5.254088	1566.45352
	21	0.164706392	135.643967	6.465241	1426.54619
	22	0.040868576	168.022686	2.562817	1385.126742
	23	0.123708676	133.94423	7.635842	1673.517493
	24	/	/	4.961204	1748.72628
				Total length = 40.784686	
04	25	/	/	/	2256.888075
	26	0.469584356	143.67508	1.816602	2294.514426
	27	0.186160232	148.775335	1.11536	2338.159116
	28	0.376474772	106.317934	0.807005	2329.941366
	29	0.483443763	77.091521	3.957948	2208.939398
	30	0.455192214	154.081453	1.350015	2246.335898
	31	0.218223989	157.6583	1.999735	2213.243098
	32	0.251269898	164.690871	3.065625	2056.32296
	33	0.55816877	113.055516	0.761886	1993.17922
	34	0.288678682	109.975108	1.086738	1961.529043
	35	/	/	0.876682	1198.739194
				Total length = 16.837595	
05	36	/	/	/	717.904477
	37	0.080638141	167.960018	2.39608	752.364732
	38	0.229839633	124.164232	2.806063	845.879221
	39	/	/	5.191562	494.095834
				Total length = 10.393705	
06	40	/	/	/	765.259158
	41	0.157368713	114.79586	8.533119	644.997759
	42	0.019954385	172.954287	4.833868	717.904477
	43	0.097934663	151.655847	7.483734	871.192165
	44	0.207736198	146.964596	2.43402	884.673463
	45	0.119425681	160.73293	3.036986	963.376641
	46	0.137283399	145.854224	2.566766	908.222604
	47	0.203439296	125.272672	5.924831	604.668624
	48	/	/	2.983212	520.315068
				Total length = 37.796536	
07	49	/	/	/	1582.895445
	50	0.311642731	123.135518	3.899045	2245.870749
	51	0.403756504	133.143854	1.999627	2187.561213
	52	0.505198692	133.682804	1.939214	2035.742986
	53	0.550931589	140.676488	1.156241	2026.129634
	54	0.48957282	146.351609	1.286363	2099.223171
	55	0.494004949	141.121857	1.077584	2156.298534
	56	0.218231813	157.088067	1.610449	2138.407969
	57	/	/	2.02918	2060.658168
				Total length = 14.997704	
08	58	/	/	/	1215.169553
	59	0.094779489	152.331999	5.48162	1211.104551
	60	0.066202292	160.550663	4.483388	1291.753221
	61	0.107370649	135.327469	5.71962	1570.004168
	62	0.146227798	135.327469	8.396156	1062.405224
	63	/	/	1.620576	556.862551
				Total length = 25.701361	
09	64	/	/	/	990.519693
	65	0.136701532	153.299749	3.719526	1425.431485
	66	/	/	3.006292	1617.612377
				Total length = 6.725819	
10	67	/	/	/	1136.209712
	68	0.464983571	131.225516	2.011128	1584.872548
	69	0.147241146	156.048879	1.53422	1759.183443
	70	/	/	4.076503	1790.453517
				Total length = 7.62185	
11	71	/	/	/	91.195824
	72	0.672652752	100.150831	1.476731	131.611181
	73	0.089054209	165.341489	2.280031	201.259025
	74	0.032663014	172.905766	3.447032	246.399081
	75	/	/	4.162382	144.408124
				Total length = 11.366176	
12	76	/	/	/	233.15457
	77	0.084902415	166.831359	2.441834	344.952127
	78	0.130846855	156.728546	2.945541	355.398408
	79	0.092547862	160.877168	3.219316	343.844458
	80	0.091633268	164.272056	3.9594	333.667342
	81	0.307659653	142.580003	2.007179	319.227414
	82	/	/	2.151165	
				Total length = 16.724436	
13	83	/	/	/	357.267262
	84	0.128197407	155.361578	1.632875	451.866843
	85	/	/	4.984406	344.952127
				Total length = 6.617281	
Maximum value		0.672652752	174.906341	8.533119	2338.159116
Minimum value		0.01960484	77.091521	0.761886	91.195824

**Table A7:** Lion Forest Garden, garden path.

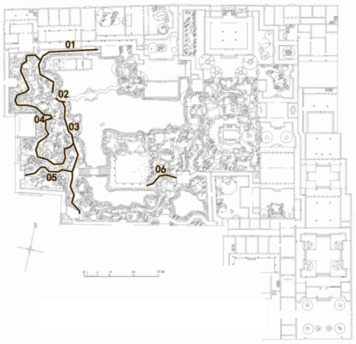

**Road No.**	**Angle No.**	**Curvature**	**Value of Angle (°)**	**Unilateral Length of Path (m)**	**Viewshed Area (m^2^)**
01	0	/	/	/	629.027353
	1	0.044480754	150.148652	19.606489	232.941613
	2	0.156841021	147.633021	3.135599	588.964084
	3	0.148508731	145.891246	3.968488	998.642445
	4	0.163755066	130.139702	3.930814	1216.460703
	5	0.20092113	114.674632	6.306005	1926.359353
	6	/	/	4.366143	1919.830684
				Total length = 41.313538	
02	7	/	/	/	2091.749924
	8	0.253594255	148.344962	1.98676	2187.34945
	9	/	/	2.314268	2202.44036
				Total length = 4.301028	
03	10	/	/	/	2153.502845
	11	0.144674607	150.19538	2.548199	2364.224476
	12	0.066815009	159.789005	4.542314	2410.252984
	13	0.139328159	149.1854	5.958943	2610.926657
	14	0.242982659	142.128111	1.572069	2578.164441
	15	0.045306152	168.546178	3.719032	2822.828661
	16	0.110132407	139.938539	5.089921	2827.021695
	17	/	/	7.323838	2808.395041
				Total length = 30.754316	
04	18	/	/	/	2844.405519
	19	0.280104788	164.042631	1.439346	2833.501835
	20	0.204363207	99.462322	1.27738	2754.26177
	21	0.080766283	171.004655	1.26	2637.429932
	22	0.206398921	149.373768	2.622251	2700.021693
	23	0.337948242	116.446065	2.495756	2722.439093
	24	0.202044851	138.522266	3.693034	1797.281935
	25	0.09939762	159.163599	3.316022	1253.534172
	26	0.280143575	121.534792	3.960114	1204.948856
	27	0.188465655	144.648707	2.991739	873.36715
	28	0.178655892	144.818724	3.450811	971.627847
	29	0.078292117	168.413677	3.315449	1229.765483
	30	0.536336673	107.315393	1.839348	1620.886258
	31	0.169471675	149.556628	2.549349	1868.478648
	32	0.382246463	105.773242	3.64055	2371.411091
	33	0.709506044	83.112447	2.62688	2462.887331
	34	0.513676027	115.983239	1.330131	2450.944186
	35	0.184859006	140.939964	2.706641	2385.721959
	36	0.123885331	150.927468	4.499	2082.989032
	37	0.064586529	165.693677	3.601694	1235.024318
	38	0.351492627	111.952452	4.11	914.909092
	39	0.323435877	138.343094	2.11322	476.776736
	40	0.266860352	130.092427	2.283725	135.780516
	41	0.181909817	150.330725	3.98995	132.852701
	42	0.198613843	116.048111	1.604307	141.030253
	43	0.116351404	142.617866	8.226912	150.511465
	44	0.284268161	138.40464	2.626082	178.531784
	45	0.693554579	101.900223	2.369219	204.734179
	46	0.578053247	138.989883	1.120045	248.014281
	47	0.465026081	111.487165	1.30292	281.857004
	48	0.249966499	131.835676	3.336615	1001.696243
	49	/	/	3.192683	996.530206
				Total length = 90.627968	
05	50	/	/	/	1130.027746
	51	0.152984354	146.380876	3.843795	1345.227836
	52	0.293083235	134.541644	3.535647	1505.130473
	53	0.397018159	131.969523	1.68	1551.184668
	54	0.253143426	134.628891	2.40753	1979.015898
	55	0.204002991	147.173336	3.663946	2586.180265
	56	0.232528313	138.308983	1.850649	2675.706206
	57	0.358179563	101.523722	4.204759	2808.395041
	58	0.077476403	166.206191	2.76	2396.69516
	59	0.178624613	142.506951	3.439186	1618.885537
	60	0.193570286	140.378362	3.75682	1122.847476
	61	0.242107789	122.466568	3.244207	969.247863
	62	0.237642362	138.388966	4.668181	579.789842
	63	/	/	1.16	450.108176
				Total length = 40.21472	
06	64	/	/	/	1787.258771
	65	0.151540228	149.283788	2.689305	1668.815669
	66	0.208574577	136.447089	4.287808	1478.196378
	67	0.109827963	158.246683	2.802089	1207.847916
	68	/	/	4.066018	1126.481101
				Total length = 13.84522	
Maximum value		0.709506044	171.004655	19.606489	2844.405519
Minimum value		0.044480754	83.112447	1.120045	132.852701

**Table A8:** Retreat and Reflection Garden, garden path.

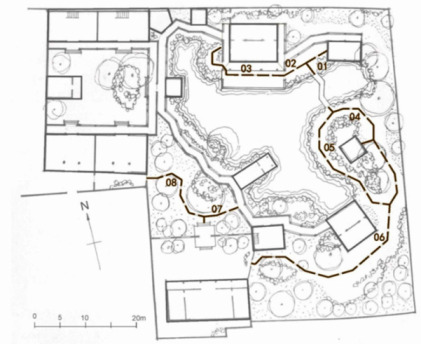

**Road No.**	**Angle No.**	**Curvature**	**Value of Angle (°)**	**Unilateral Length of Path (m)**	**Viewshed Area (m^2^)**
01	0	/	/	/	1194.10
	1	0.539413144	82.905634	4.9197155	1527.53
	2	/	/	2.842654	1536.11
				Total length = 7.7623695	
02	3	/	/	/	1536.02
	4	0.165866618	97.094371	2.8506145	1527.53
	5	/	/	4.9197155	1533.41
				Total length = 7.77033	
03	6	/	/	/	907.84
	7	0.23185141	120.398099	1.9988655	1279.55
	8	0.308933141	96.375739	2.285097	1568.20
	9	0.246620998	132.56953	2.024689	1704.42
	10	0.295874378	143.806209	1.2175915	1598.95
	11	0.086918986	90.70563	0.8792465	1596.86
	12	/	/	11.459616	1533.41
				Total length = 19.8651055	
04	13	/	/	/	977.91
	14	0.099242873	137.592342	2.911386	1433.03
	15	0.108844732	132.407658	4.355949	1548.75
	16	0.062553802	162.573957	3.0351115	1543.22
	17	0.307814368	115.304673	2.081357	1548.31
	18	/	/	1.365382	1553.55
				Total length = 13.749186	
05	19	/	/	/	1548.31
	20	0.080973845	141.47629	4.3446315	1550.54
	21	0.068701128	145.455295	3.80106	1523.47
	22	0.048559095	150.279672	4.836567	1538.64
	23	0.053507926	147.157236	5.7237605	1284.55
	24	0.105011529	128.264777	4.8397565	1056.70
	25	/	/	3.44192	1123.46
				Total length = 26.987695	
06	26	/	/	/	659.98
	27	0.340654461	121.165923	0.793246	663.76
	28	0.117225041	134.164444	2.008021	636.95
	29	0.065166117	144.628597	4.5484765	1306.29
	30	0.036895761	159.837398	4.774853	1271.39
	31	0.055242282	149.02702	4.713563	1179.27
	32	0.03102133	154.852902	4.953099	989.00
	33	0.047892631	134.694756	9.052118	926.94
	34	0.048857981	152.162708	7.008921	1123.46
	35	0.101835005	141.112251	2.7816135	1270.48
	36	0.036927116	155.190506	3.7470515	1183.63
	37	0.109295799	104.792452	7.85266	977.91
	38	/	/	2.535031	691.85
				Total length = 54.768654	
07	39	/	/	/	1373.24
	40	0.036283443	165.310415	2.5480035	1262.58
	41	/	/	4.494537	1134.73
				Total length = 7.0425405	
08	42	/	/	/	1448.89
	43	0.060123669	162.343635	2.101492	1467.85
	44	0.158974864	129.843921	3.0018815	1449.07
	45	0.194818214	116.731607	2.320937	1469.24
	46	0.080214298	147.798762	3.0449795	1435.75
	47	0.120990886	130.571453	3.865482	1413.23
	48	0.27121138	105.256395	3.035094	1373.24
	49	/	/	1.2213	1367.82
				Total length = 18.5911655	
Maximum value		0.539413144	165.310415	11.459616	1704.42
Minimum value		0.03102133	82.905634	0.793246	636.95

**Table A9:** Couple’s Garden Retreat, garden path.

Part1		The East Garden of Couple’s Garden Retreat			

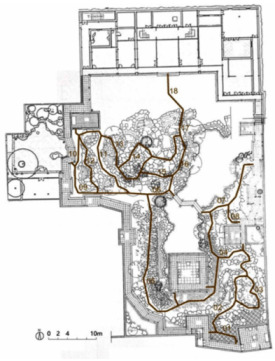

**Road No.**	**Angle No.**	**Curvature**	**Value of Angle (°)**	**Unilateral Length of Path (m)**	**Viewshed Area (m^2^)**
01	0	/	/	/	442.113539
	1	0.58560936	151.60821	0.706873	470.857816
	2	0.361975161	125.028533	0.967013	509.387689
	3	0.100683075	163.625066	3.899616	590.967735
	4	/	/	1.749648	503.89532
				Total length = 7.323151	
02	5	/	/	/	302.858801
	6	0.463032286	136.855828	1.299594	382.637432
	7	0.407871953	146.345705	1.848907	476.849889
	8	0.237493417	95.506198	0.978288	614.016874
	9	0.426692242	155.319606	1.18666	701.32275
	10	0.263168837	130.029907	0.815477	722.351556
	11	0.216404846	129.94909	0.514547	727.72085
	12	0.587336469	136.784638	1.586157	701.433415
	13	0.062318576	86.991638	0.907082	642.851711
	14	0.355129385	152.799999	1.695105	563.24478
	15	0.022656072	129.14705	0.946806	511.035828
	16	0.397263512	155.927703	0.729594	512.691088
	17	0.324453158	156.691661	1.365113	541.179613
	18	0.116447861	171.190745	1.124935	561.104062
	19	0.300590207	156.591519	1.51334	559.346099
	20	0.361536239	158.306323	1.185008	576.10674
	21	0.238814475	165.577577	0.8964	615.913258
	22	0.016415521	178.578245	1.205892	634.334424
	23	0.233620665	154.792866	1.820449	633.880383
	24	0.460065638	122.686065	1.915701	610.314907
	25	/	/	2.249767	590.967735
				Total length = 25.784822	
03	26	/	/	/	559.346099
	27	0.211807383	146.828962	3.164679	492.537572
	28	0.244761431	89.355665	2.218444	503.128809
	29	0.239327322	159.709419	1.47102	517.580613
	30	0.558952361	133.125898	1.472495	544.698795
	31	0.087385417	81.77282	1.373613	521.372933
	32	0.616795185	124.783657	1.886583	601.887902
	33	0.321406001	157.659757	1.089746	624.082384
	34	/	/	1.320412	642.851711
				Total length = 13.996991	
04	35	/	/	/	382.637432
	36	0.376775861	139.950733	2.09774	702.326264
	37	0.471408686	126.978414	1.531999	797.307959
	38	/	/	2.239105	982.034997
				Total length = 5.868845	
05	39	/	/	/	1138.942188
	40	0.225573011	147.495511	2.597224	1163.5089
	41	0.16743333	148.842916	2.364613	1186.373208
	42	0.227051953	141.291939	4.034274	1126.338667
	43	0.187993917	124.67373	1.748295	982.034997
	44	0.147172528	133.869813	7.635566	653.374423
	45	0.204206856	143.853878	2.812732	367.58578
	46	0.098610875	156.074469	3.262132	308.532356
	47	0.116914705	156.263473	5.136563	600.464534
	48	0.346268081	134.985187	1.866876	622.227403
	49	0.148172225	145.407048	2.545993	698.629843
	50	0.127507831	149.67738	5.430085	774.653054
	51	0.034350537	166.039011	2.742273	840.123098
	52	0.034842074	164.16839	11.370804	1097.6156
	53	/	/	4.410437	1027.014302
				Total length = 57.957867	
06	54	/	/	/	1078.271105
	55	0.181698379	97.991276	1.620213	1033.991519
	56	0.213026731	149.694733	1.586819	1043.782163
	57	0.026541542	174.59687	3.299181	1025.734083
	58	/	/	3.803356	1037.069428
				Total length = 10.309568	
07	59	/	/	/	1144.077125
	60	0.296900667	147.132571	1.856318	1139.114668
	61	0.182397887	157.338896	1.955049	1084.243415
	62	0.268872638	144.142187	2.352803	944.088681
	63	0.163153667	156.149417	2.226753	909.727853
	64	0.033126675	173.178928	2.837631	883.42929
	65	0.376121049	103.412384	4.344896	850.552486
	66	/	/	1.973968	833.662627
				Total length = 17.547419	
08	67	/	/	/	944.088681
	68	0.314873703	142.715684	2.725636	949.245169
	69	0.610114282	124.345093	1.307247	914.553625
	70	/	/	1.744285	871.190432
				Total length = 5.777168	
09	71	/	/	/	1037.069428
	72	0.158131078	114.032686	10.799553	871.068565
	73	0.136761495	156.810278	1.614151	912.236592
	74	0.259424211	137.925199	4.239821	915.524847
	75	/	/	1.167503	901.441424
				Total length = 17.821029	
10	76	/	/	/	442.10357
	77	0.082673818	126.939691	0.819936	524.900351
	78	0.276296241	123.683394	0.773813	584.011319
	79	0.144907181	150.761954	5.55959	880.988313
	80	/	/	1.318735	915.524847
				Total length = 8.472074	
11	81	/	/	/	584.011319
	82	0.144018277	161.696393	1.930995	540.363155
	83	0.604069319	112.499415	2.485073	758.579787
	84	0.381343925	137.195292	1.070275	748.42619
	85	0.163044099	155.768698	2.703569	788.877161
	86	0.082062411	169.27985	2.44527	884.007121
	87	0.242413523	150.519528	2.108603	903.656398
	88	0.20952462	160.021029	2.089783	969.01058
	89	0.493782317	130.557027	1.217707	1026.213595
	90	0.408182159	132.603869	2.142992	1034.656364
	91	0.277567151	157.414838	1.792762	1035.485423
	92	/	/	1.025426	1037.069428
				Total length = 21.012456	
12	93	/	/	/	912.851621
	94	0.562563404	125.612861	1.033892	921.091154
	95	0.325351841	133.435637	2.163127	961.37132
	96	0.403165939	126.071341	2.691017	929.115087
	97	0.422240272	118.276112	1.784184	899.41032
	98	0.311120268	134.359611	3.019084	823.802943
	99	0.13358365	166.238427	1.946809	731.565006
	100	/	/	1.640075	584.02886
				Total length = 14.278189	
13	101	/	/	/	1522.699024
	102	0.309015171	128.600594	2.069051	1578.156484
	103	0.140867149	155.084626	3.501793	1605.975349
	104	0.2100333	142.284299	2.62036	1590.777103
	105	0.317616322	123.501955	3.527132	1611.983708
	106	0.349280911	115.17193	2.403006	1622.291898
	107	0.153342044	154.383207	3.681694	1632.640473
	108	/	/	2.089574	1636.882685
				Total length = 19.892611	
14	109	/	/	/	1594.766087
	110	0.255637959	147.763248	2.922068	1627.495161
	111	0.136062615	164.242515	1.399937	1630.52187
	112	0.529762293	90.000005	2.626575	1635.127255
	113	0.265094351	147.713605	2.711806	1632.148078
	114	0.327553922	124.697975	1.468042	1636.882685
	115	0.357471834	113.164075	4.037026	1632.884289
	116	0.364194704	143.649075	1.973196	1614.717621
	117	0.093312681	170.518382	1.448501	1619.019854
	118	0.198328948	87.414424	2.093723	1612.107732
	119	/	/	1.449662	1604.235896
				Total length = 22.130537	
15	120	/	/	/	696.455467
	121	0.101489488	165.834232	3.384675	754.685825
	122	0.308499066	147.190802	1.468249	793.864681
	123	/	/	2.232766	738.148932
				Total length = 7.08569	
16	124	/	/	/	1604.22293
	125	0.166047203	142.446619	2.864873	1616.494426
	126	0.145883263	152.027465	4.859473	1618.707401
	127	0.216153688	148.670794	1.720925	1623.70731
	128	/	/	3.256973	1626.44239
				Total length = 12.702245	
17	129	/	/	/	1604.22293
	130	0.155103441	161.113008	1.412904	1610.805894
	131	/	/	4.275733	1617.165222
				Total length = 5.688637	
18	132	/	/	/	721.485888
	133	0.114980822	139.395987	4.628519	673.799223
	134	/	/	4.206704	713.935361
				Total length = 8.835223	
**Part2**		**The west garden of Couple’s Garden Retreat**			

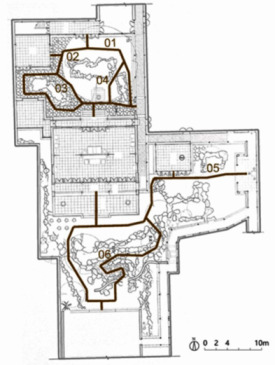

**Road No.**	**Angle No.**	**Curvature**	**Value of Angle (°)**	**Unilateral length of path (m)**	**Viewshed area (m^2^)**
01	0	/	/	/	207.073488
	1	0.261535145	115.745608	4.589999	234.925415
	2	0.080430967	160.686015	3.5155	251.64025
	3	/	/	4.824186	237.47987
				Total length = 12.929685	
02	4	/	/	/	207.073488
	5	0.326273945	111.464503	3.505662	195.913969
	6	0.290533578	106.340276	3.397029	211.446141
	7	0.363324242	89.760363	4.789756	167.368386
	8	0.486648554	90.239638	2.73275	155.052295
	9	0.346915337	120.346541	3.05797	208.878099
	10	0.346592991	125.408083	2.672578	198.340465
	11	0.331188052	138.109209	2.620038	206.306636
	12	0.218064703	142.004488	1.682504	209.808627
	13	0.04938536	170.150896	4.224459	214.083918
	14	0.107841659	168.984685	2.726003	236.130276
	15	/	/	0.828114	267.325693
				Total length = 32.236863	
03	16	/	/	/	211.446141
	17	0.19323015	155.587474	2.599429	212.092276
	18	0.166766106	165.307253	1.773313	211.65634
	19	/	/	1.292552	209.808627
				Total length = 5.665295	
04	20	/	/	/	243.925468
	21	0.163931194	146.767977	4.072695	216.02169
	22	/	/	2.509541	213.427258
				Total length = 6.582236	
05	23	/	/	/	337.874407
	24	0.054375966	171.018819	3.059639	343.351845
	25	0.033927425	169.958908	2.700371	343.421178
	26	0.22938451	112.13876	7.609361	343.469639
	27	0.215214927	156.801315	1.075413	364.300611
	28	0.159793703	154.83471	2.647294	376.79655
	29	0.349969835	126.014981	2.805975	367.139996
	30	0.037192031	169.150306	2.377101	319.667695
	31	0.20647535	114.412617	7.776702	322.122986
	32	0.175218362	155.63872	2.044529	325.191025
	33	0.129794478	150.461334	2.769287	134.838456
	34	0.151758943	150.512672	5.063987	103.316179
	35	0.756451861	91.530752	1.581162	132.900405
	36	0.805694658	91.62621	2.076097	103.246189
	37	/	/	1.230967	109.494436
				Total length = 44.817884	
06	38	/	/	/	486.176777
	39	0.386272206	118.37427	3.159153	487.967704
	40	0.328284285	138.137434	2.108097	473.031483
	41	0.184400054	148.809957	2.244516	472.378633
	42	0.176134117	146.600998	3.575342	444.056169
	43	0.643608138	81.419903	2.947805	444.619157
	44	0.585591903	111.811537	1.412007	439.461277
	45	0.772438956	89.999984	2.362601	430.704673
	46	0.736787217	114.034962	1.05927	420.178755
	47	0.388588558	131.111811	1.851025	372.796905
	48	0.638528503	89.999973	2.40121	146.612728
	49	/	/	2.011547	193.180624
				Total length = 25.132574	
Maximum value		0.805694658	178.578245	11.370804	1636.882685
Minimum value		0.016415521	58.362688	0.514547	103.246189

**Table A10:** Jichang Garden, garden path.

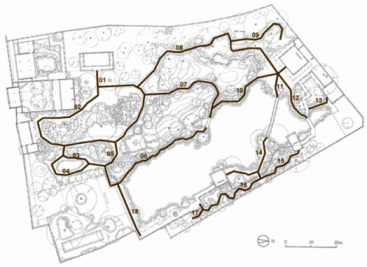

**Road No.**	**Angle No.**	**Curvature**	**Value of Angle (°)**	**Unilateral Length of Path (m)**	**Viewshed Area (m^2^)**
01	0	/	/	/	1524.045037
	1	6.5287 × 10^−6^	179.950152	4.057847	2126.73176
	2	0.140159623	90.000005	2.372756	2231.770295
	3	0.058903472	149.845627	14.070759	2065.484893
	4	0.175597973	149.477923	3.356151	2041.451097
	5	0.179208307	140.139261	2.636278	2042.428938
	6	0.049605146	164.482757	4.927066	2057.863303
	7	0.071205238	156.76815	5.957211	2050.593959
	8	/	/	5.353356	2082.602855
				Total length = 42.731423	
02	9	/	/	/	2231.770295
	10	0.215856677	123.592885	3.369485	2306.24649
	11	0.052249755	166.836477	5.325157	2400.940611
	12	0.069066118	161.937815	3.446623	2542.131741
	13	0.092858628	153.327777	5.637764	2402.686226
	14	0.015937788	175.27576	4.292974	1970.731888
	15	0.136066692	145.235884	6.051585	1807.073994
	16	0.246286824	110.774895	2.666452	1829.307101
	17	0.124617307	139.124043	6.225833	2297.492759
	18	0.155148927	147.791385	4.973064	2565.618709
	19	0.063086918	170.945216	2.131098	2604.487882
	20	0.076234606	157.958075	2.872725	2669.83579
	21	0.00526166	177.833193	7.122986	2814.25546
	22	0.039886657	164.885232	7.248137	2745.075414
	23	0.146112232	136.851216	5.940068	2447.436829
	24	0.133172344	153.312288	4.099984	2078.359748
	25	/	/	2.825646	2082.602855
				Total length = 74.22958	
03	26	/	/	/	2669.83579
	27	0.373457494	111.586523	3.892348	2737.105522
	28	0.219096826	151.306445	1.988319	2851.003115
	29	0.036492212	172.61771	2.533176	2917.816628
	30	0.214659382	136.934381	4.522455	3215.752298
	31	/	/	2.258419	3473.262689
				Total length = 15.194718	
04	32	/	/	/	2737.105522
	33	0.245548346	103.087296	5.77181	2711.147023
	34	0.238543907	121.263774	4.290802	2936.827662
	35	0.081314544	155.481307	3.930193	3037.487368
	36	0.080819393	155.703355	6.500429	3326.144924
	37	0.165283614	129.890151	3.899855	3473.262689
	38	/	/	6.287706	3738.683524
				Total length = 30.680794	
05	39	/	/	/	2447.436829
	40	0.161829058	139.272239	4.792882	2985.103569
	41	0.14857324	149.468129	3.800649	3839.309307
	42	/	/	3.286554	3738.683524
				Total length = 11.880086	
06	43	/	/	/	3738.683524
	44	0.018617878	175.547165	3.129872	3896.201775
	45	0.306588988	89.074477	5.216066	3114.385681
	46	0.184384767	145.482355	4.001193	3606.885774
	47	1.11924 × 10^−5^	179.999967	2.41657	3649.361177
	48	0.060882384	170.920022	3.110193	3613.187847
	49	0.116591798	165.769023	2.089932	3482.352448
	50	0.244877633	142.31064	2.160448	3329.375732
	51	0.163119158	159.479448	3.106126	3194.820384
	52	0.280999907	142.713847	1.248517	3208.107122
	53	0.290259091	142.713847	3.251521	3044.940351
	54	0.473051653	139.681382	1.093183	3065.742524
	55	0.331015955	151.646465	1.80927	3025.815298
	56	0.49690568	131.065316	1.145024	2891.685814
	57	0.091912031	163.94809	2.156916	2950.894663
	58	0.119997855	162.187783	3.914626	2930.908262
	59	0.306105071	139.919175	1.228165	2706.082681
	60	0.241432057	146.876423	3.191826	3238.454983
	61	0.329583352	136.223244	1.504093	3405.427255
	62	0.181675985	151.344231	2.981268	3381.421355
	63	0.349569477	127.170281	2.465925	3334.385326
	64	0.407913235	124.479076	2.623866	3465.326151
	65	/	/	1.92877	3417.438274
				Total length = 55.773372	
07	66	/	/	/	3271.483329
	67	0.094836131	162.019064	2.237377	3260.431856
	68	0.232447154	127.915084	4.34545	3348.406676
	69	0.177909248	152.476907	3.188187	2427.337647
	70	0.150760206	148.318254	2.154251	1972.272026
	71	0.134107023	151.824189	5.042006	1045.828021
	72	0.218360863	147.515319	2.182318	721.812708
	73	0.239791477	127.744166	2.936677	474.662568
	74	0.121194095	150.923606	4.375408	645.188768
	75	0.066136633	153.874759	3.908829	1060.027817
	76	/	/	9.694558	2042.428938
				Total length = 40.06506	
08	77	/	/	/	2063.64496
	78	0.045752652	160.497251	9.499521	2006.60357
	79	0.067695851	161.812339	5.290155	2238.087207
	80	0.19443003	128.21388	4.046328	2405.108186
	81	0.058481446	157.20185	4.927717	1114.585539
	82	0.056107946	162.710508	8.571181	2613.017976
	83	0.148923693	162.039515	2.099238	2783.189346
	84	0.311419684	136.45071	2.093608	2952.936842
	85	0.161482688	138.949029	2.665394	3050.60645
	86	0.296537338	88.622848	5.932856	3025.782012
	87	0.103368939	160.918348	3.349442	3233.900351
	88	0.304528353	141.494451	3.064379	3315.30368
	89	0.251297411	118.010499	1.218494	3398.938723
	90	0.033452857	169.50495	6.371393	3794.949434
	91	0.107204389	124.70564	4.563585	3849.948849
	92	/	/	12.27254	3727.352949
				Total length = 75.965831	
09	93	/	/	/	3238.633181
	94	0.042641245	167.917598	6.260732	3672.144719
	95	0.126380274	158.805292	3.607914	3847.722431
	96	0.29322999	138.840344	2.206689	3570.842705
	97	0.061939508	166.384043	2.586238	3020.254036
	98	0.239591325	127.244301	5.063103	3279.219934
	99	0.366437544	132.41676	2.219138	3185.494829
	100	0.236785715	134.183855	2.184227	2892.506722
	101	/	/	4.32899	3268.854601
				Total length = 28.457033	
10	102	/	/	/	3381.422258
	103	0.166498504	137.943823	3.737883	3271.483329
	104	0.108440499	143.769637	4.871492	3451.951705
	105	0.162834886	130.733791	6.583806	3645.304234
	106	0.17313121	145.944077	3.560675	3682.605347
	107	0.169565914	133.826088	3.204122	3688.081256
	108	0.062215383	154.47555	5.97069	3757.123037
	109	/	/	8.222546	3731.893649
				Total length = 36.151214	
11	110	/	/	/	3731.893649
	111	0.082204165	157.054161	5.569367	3762.101446
	112	/	/	4.104504	3711.726219
				Total length = 9.673871	
12	113	/	/	/	3545.39741
	114	0.452107026	140.819404	0.864186	3582.680403
	115	0.144766008	156.003424	2.070909	3676.67015
	116	0.12053686	157.615601	3.662705	3681.677563
	117	0.087600677	161.382101	2.776323	3665.582939
	118	0.088367715	146.748319	4.604046	3665.80908
	119	0.209876067	93.291022	8.300535	3731.893649
	120	/	/	4.093041	3530.711469
				Total length = 26.371745	
13	121	/	/	/	3380.245225
	122	0.218950996	109.767769	2.060373	3301.091549
	123	/	/	7.629966	3555.598118
				Total length = 9.690339	
14	124	/	/	/	2236.138916
	125	0.148130104	145.784838	3.381498	2470.629937
	126	0.075025727	166.946826	4.471437	2476.599614
	127	0.270605849	135.353586	1.579637	2441.243737
	128	0.138655442	132.016799	3.950011	2626.844566
	129	0.080790434	151.208363	7.662754	3409.795604
	130	0.207913263	136.540649	4.621727	3577.616602
	131	/	/	2.448065	3611.36196
				Total length = 28.115128	
15	132	/	/	/	1085.970097
	133	0.046523407	174.077405	2.192162	425.316338
	134	0.015450532	178.28863	2.248316	429.008328
	135	0.499639722	121.608737	1.612188	430.450165
	136	0.385776476	117.629325	2.275281	394.200751
	137	0.142355055	157.821984	3.07213	410.167306
	138	0.226416709	148.562324	2.330417	413.698559
	139	0.297926067	131.123426	2.455531	311.799709
	140	0.399629883	124.059417	3.091513	382.206929
	141	/	/	1.418815	241.785404
				Total length = 20.696354	
16	142	/	/	/	2607.530771
	143	0.392327757	96.687978	3.078102	1085.970097
	144	0.217273917	141.45698	3.67743	370.777429
	145	0.333428113	136.169957	2.381206	429.797496
	146	0.301571957	139.40019	2.09468	406.481432
	147	0.433985245	113.678461	2.504557	341.603887
	148	0.253686456	145.27467	2.536889	400.628501
	149	0.662688938	90.000004	2.16694	422.852239
	150	0.452372777	128.173378	2.100728	410.667079
	151	0.49495417	121.103862	1.759871	343.895914
	152	0.367898446	106.912219	2.205284	209.985126
	153	0.27400109	122.131397	4.112438	457.241205
	154	0.327257302	131.502956	2.91929	467.316147
	155	0.591286237	102.610677	2.086514	1304.320152
	156	0.319350378	135.137832	2.14249	1495.350142
	157	/	/	2.632733	1569.488678
				Total length = 38.399153	
17	158	/	/	/	1495.350142
	159	0.131617538	168.451569	0.942473	576.355135
	160	0.263066518	141.85931	2.113942	375.021395
	161	/	/	2.847896	371.845013
				Total length = 5.90431	
18	162	/	/	/	1569.488678
	163	0.21312662	92.067961	3.154652	2781.112548
	164	/	/	8.631469	1474.523161
				Total length = 11,786121	
Maximum value		0.662688938	179.999967	14.070759	3896.201775
Minimum value		6.5287 × 10^−6^	88.622848	0.864186	209.985126

**Table A11:** Humble Administrator’s Garden, corridor.

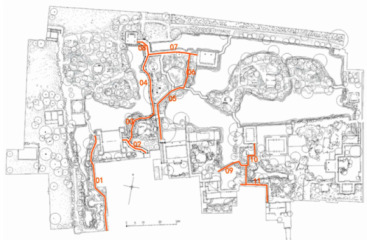

**Road No.**	**Angle No.**	**Curvature**	**Value of Angle (°)**	**Unilateral Length of Path (m)**	**Viewshed Area (m^2^)**
01	0	/	/	/	1591.897134
	1	0.280035127	147.204736	9.242218	1512.227602
	2	0.105712784	149.621176	4.010587	1570.787319
	3	0.051348388	162.719927	6.794937	1295.962864
	4	0.153615863	143.906818	7.842136	2590.791263
	5	0.051679046	155.106654	13.601001	2737.350019
	6	0.067422197	137.380002	2.913648	2924.577844
	7	0.099676909	137.198631	5.120852	2761.487571
	8	0.043595305	166.467099	6.579567	2407.561215
	9	0.040707357	164.475035	3.294296	2373.029683
	10	/	/	6.6304	2431.119518
				Total length = 66.029642	
02	11	/	/	/	2866.375249
	12	0.32695333	121.933609	3.319335	3207.599312
	13	0.097597147	159.01901	3.553329	2800.105012
	14	0.116873818	154.375347	3.908584	2259.959361
	15	0.128670027	155.631441	3.680904	1747.904378
	16	0.524092756	125.849637	3.87798	1817.816697
	17	/	/	3.4573	1880.802559
				Total length = 21.797432	
03	18	/	/	/	3919.72128
	19	0.149280005	146.611715	3.951841	3417.279949
	20	0.090216097	161.709653	3.745194	3940.624248
	21	0.032044231	174.11362	5.301367	4530.673907
	22	0.24427801	131.49227	3.10799	4832.187012
	23	0.107895271	159.273744	4.614578	4914.076687
	24	0.084647698	167.097757	3.053545	5264.479539
	25	0.142872498	155.692833	2.255013	5439.260311
	26	/	/	3.631811	6164.850143
				Total length = 29.661339	
04	27	/	/	/	5819.134909
	28	0.100786368	151.551651	8.274105	5611.713876
	29	0.261080542	139.732262	3.171	5596.804697
	30	0.123284173	154.054071	3.872693	5514.674225
	31	0.084616484	163.29568	3.410225	5674.773421
	32	0.028498282	174.25378	3.456379	5494.773644
	33	0.039344607	171.713409	3.579017	5390.541749
	34	0.032667538	173.002825	3.76644	5824.471538
	35	0.019924566	174.468854	3.705684	5867.772748
	36	/	/	5.980153	6064.850143
				Total length = 39.215696	
05	37	/	/	/	2881.984343
	38	0.007560937	176.815089	11.881391	4982.504641
	39	0.012563647	176.738247	5.09221	6083.21067
	40	0.178341163	130.227385	9.242132	6161.206926
	41	0.00967491	176.792066	3.081833	6193.829177
	42	0.051885665	159.347695	8.489747	6191.266459
	43	0.129681192	151.117937	5.317096	6290.212098
	44	/	/	2.336723	6298.226974
				Total length = 45.441132	
06	45	/	/	/	6098.226974
	46	0.102576117	150.794768	7.302973	5496.698232
	47	0.139183927	155.714768	2.497418	5408.594311
	48	0.045834584	170.79801	3.502815	4765.219533
	49	0.030762517	173.186646	3.49768	4569.174428
	50	/	/	4.22883	4952.970734
				Total length = 21.029716	
07	51	/	/	/	5052.970734
	52	0.004524436	174.793697	3.968165	5420.022574
	53	0.022340768	171.153464	4.680738	5867.789383
	54	0.013929986	177.879203	9.123622	5960.647574
	55	/	/	7.237596	6219.134909
				Total length = 25.010121	
08	56	/	/	/	6119.134909
	57	0.194318957	136.630218	6.263467	5912.345427
	58	0.164733116	141.951262	4.68673	5338.98149
	59	/	/	4.861997	5026.056833
				Total length = 15.812194	
09	60	/	/	/	2056.834522
	61	0.032494024	167.283068	12.180595	2080.799054
	62	0.400164154	103.706964	5.431729	2104.465612
	63	0.36604715	118.000364	3.716905	2136.537328
	64	/	/	3.921239	2399.417803
				Total length = 25.250468	
10	65	/	/	/	2299.284383
	66	0.338669311	89.692108	5.50092	2431.180131
	67	0.280814394	90	5.74901	2660.600207
	68	0.248022234	90	4.20402	3508.395585
	69	/	/	6.886923	2497.036695
				Total length = 22.340873	
11	70	/	/	/	1843.308193
	71	0.106433252	168.347937	3.42521	2054.322063
	72	0.026282445	170.050668	5.88229	1908.101874
	73	0.125070556	93.95866	11.778187	2148.277887
	74	/	/	9.977054	1829.775396
				Total length = 31.062741	
Maximum value		0.524092756	177.879203	13.601001	6298.226974
Minimum value		0.004524436	89.692108	2.255013	1295.962864

**Table A12:** Lingering Garden, corridor.

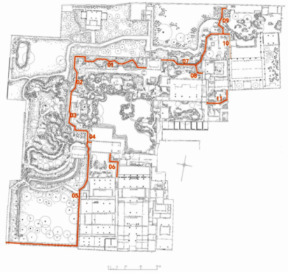

**Road No.**	**Angle No.**	**Curvature**	**Value of Angle (°)**	**Unilateral Length of Path (m)**	**Viewshed Area (m^2^)**
01	0	/	/	/	1684.974021
	1	0.253352106	116.689974	5.523113	1483.086723
	2	0.104132238	163.318775	2.558539	1383.767139
	3	0.241217209	107.517207	3.013116	1240.562498
	4	0.101988059	153.369417	6.459277	1006.035987
	5	0.201118325	146.627363	2.525411	877.117722
	6	0.261641729	130.847234	3.181903	852.680258
	7	0.252113995	136.082001	3.176502	810.875375
	8	0.125167801	150.757765	2.753974	836.790495
	9	0.15464744	143.007282	5.285751	843.936002
	10	0.158656769	142.692263	2.880319	846.543348
	11	0.347407522	89.665066	5.147245	1066.208088
	12	0.294605131	89.805471	2.608432	1107.204503
	13	/	/	6.27645	1293.495926
				Total length = 51.390029	
02	14	/	/	/	1293.495926
	15	0.108342838	146.249971	7.183382	1673.624517
	16	0.097651381	142.918504	3.474682	1762.163351
	17	0.100243228	142.127707	9.398001	1679.800482
	18	0.136252705	140.203727	3.385726	1055.078818
	19	/	/	6.540504	1165.225428
				Total length = 29.982296	
03	20	/	/	/	2169.726342
	21	0.257271948	93.386618	7.297772	1814.500561
	22	0.294657029	121.34754	2.242959	1975.138971
	23	0.234913114	110.950183	4.30426	1875.617377
	24	0.138580522	150.364933	5.320983	1848.713512
	25	0.115359879	155.037643	2.00857	1861.276386
	26	/	/	5.446417	1165.225428
				Total length = 26.620962	
04	27	/	/	/	2169.726342
	28	0.203807151	135.1345	0.957593	2185.135442
	29	0.295559362	91.274883	6.210923	1002.135053
	30	/	/	2.547117	
				Total length = 9.715633	
05	31	/	/	/	1516.093508
	32	0.012665468	179.148487	1.268396	1044.16427
	33	0.461852471	134.444926	1.078298	1227.820238
	34	0.455352004	121.328631	2.239138	952.630178
	35	0.256840593	145.322233	2.063438	814.672416
	36	0.033239718	163.073644	2.575177	1736.719122
	37	0.041173898	156.565597	15.038067	2694.594861
	38	0.123108262	152.479252	4.571261	2745.900043
	39	/	/	3.149431	3279.972007
				Total length = 31.983205	
06	40	/	/	/	198.697178
	41	0.281441993	89.999762	4.9394	245.297039
	42	0.188424791	89.324921	5.108357	197.213641
	43	/	/	9.363753	240.440588
				Total length = 19.41151	
07	44	/	/	/	299.554023
	45	0.128426513	137.502016	7.626173	227.005006
	46	0.100841855	137.255607	3.550057	1650.033084
	47	0.00545464	178.22716	10.636731	1855.717688
	48	0.268512304	142.956739	3.902565	1891.436651
	49	0.174442062	142.191583	3.525412	2007.774305
	50	0.086263985	163.941277	2.950971	2141.764948
	51	0.073933033	155.087771	8.650493	2323.116726
	52	0.125072649	134.660025	3.485615	2349.495198
	53	0.158400346	141.634791	4.799245	2365.844325
	54	/	/	7.403294	2389.828806
				Total length = 56.530556	
08	55	/	/	/	1271.556902
	56	0.292529689	150.550589	2.671249	1485.891579
	57	0.275787435	132.887552	0.768118	1544.184704
	58	/	/	4.760805	1879.016518
				Total length = 8.200173	
09	59	/	/	/	1733.046342
	60	0.325971937	90.333967	2.529417	2305.495822
	61	0.142407051	146.653281	5.57031	2450.164327
	62	0.132446302	159.855455	2.433766	2455.326782
	63	0.377664468	109.683627	2.84757	2444.710211
	64	0.272456295	124.804959	3.24492	2425.845951
	65	/	/	3.55436	2286.93438
				Total length = 20.180343	
10	66	/	/	/	2110.603353
	67	0.004571687	178.928226	4.151885	983.783778
	68	0.000253193	179.920168	4.031444	744.918239
	69	/	/	6.975388	663.9331
				Total length = 15.158717	
11	70	/	/	/	101.359089
	71	0.316981625	92.404698	6.240973	99.355264
	72	0.206900606	153.769493	0.664807	100.097964
	73	0.059452968	172.582397	3.665959	102.068613
	74	0.091612621	157.61662	0.681775	104.194026
	75	0.260026212	88.119789	7.678858	77.14784
	76	/	/	0.693075	15.031317
				Total length = 19.625448	
Maximum value		0.461852471	179.999999	15.038067	3279.972007
Minimum value		0.000253193	88.119789	0.664807	15.031317

**Table A13:** Canglang Pavilion, corridor.

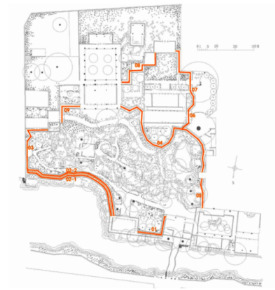

**Road No.**	**Angle No.**	**Curvature**	**Value of Angle (°)**	**Unilateral Length of Path (m)**	**Viewshed Area (m^2^)**
01	0	/	/	/	963.98553
	1	0.202407881	89.205105	0.716039	1641.727207
	2	0.121047862	93.113012	9.845798	928.855909
	3	/	/	12.674381	840.870631
				Total length = 23.236217	
02-1	4	/	/	/	1089.865495
	5	0.00897996	176.542921	9.827962	1868.329556
	6	0.121775	157.030885	3.263618	1883.637451
	7	0.0818092	164.398277	3.336654	1882.940469
	8	0.0408673	173.42901	3.321645	1895.00276
	9	0.0231998	174.066226	3.279517	1899.092785
	10	0.0899515	164.255535	3.277099	1915.254383
	11	0.0766299	163.962386	3.093499	1934.6468
	12	0.0353728	172.034438	4.249876	1952.843233
	13	0.130972	150.451247	3.558809	1971.369268
	14	0.0758709	147.754465	4.158761	2054.702217
	15	0.0804704	147.545717	10.371151	2076.254352
	16	0.147235	146.90809	3.36916	1723.467919
	17	0.0994901	165.642198	4.362092	473.114366
	18	/	/	0.64031	665.71501
				Total length = 60.110152	
02-2	19	/	/	/	872.083064
	20	0.0212947	171.434439	9.977502	1073.706791
	21	0.0871852	165.531079	3.728006	1042.729125
	22	0.073888	159.851686	3.15231	964.180858
	23	0.0332146	174.733897	4.136272	866.825264
	24	0.0390372	171.79779	3.434574	900.248717
	25	0.0728386	165.761194	3.606294	875.179377
	26	0.0762772	162.065452	3.557942	557.852837
	27	0.0514917	168.216432	4.426894	405.504411
	28	0.136772	149.032214	3.526935	471.863659
	29	0.0778663	149.142848	4.310711	468.917301
	30	0.090443	145.647261	9.363313	704.827345
	31	0.140138	146.105653	3.549355	813.685659
	32	/	/	5.005657	841.956635
				Total length = 61.775765	
03	33	/	/	/	841.956635
	34	0.022108282	171.920515	11.100002	884.63924
	35	0.005771177	177.594294	3.448886	987.922805
	36	0.193096366	143.017712	3.120135	1017.838042
	37	0.314000184	113.874047	3.814942	950.500968
	38	0.185537754	133.026722	7.3024	518.389007
	39	/	/	3.3015	356.348942
				Total length = 32.087865	
04	40	/	/	/	1103.162748
	41	0.184030488	127.08085	5.207426	1732.339367
	42	0.26857041	114.245071	4.42919	1213.667548
	43	0.190105756	125.403325	3.639631	1215.232752
	44	0.054190998	164.070176	5.937107	2057.183906
	45	0.001114635	179.830519	4.288306	2157.542669
	46	0.087887074	165.778723	1.018875	2121.184757
	47	0.101117153	154.871156	3.597269	1952.679836
	48	0.084686983	158.489867	3.007083	1873.117747
	49	0.178624004	130.579465	3.805758	1946.194432
	50	0.107287253	152.549576	4.554639	2047.396099
	51	0.086211788	158.67715	4.291127	2036.157016
	52	0.250544868	89.999998	4.292617	1870.549615
	53	0.069381585	150.299271	6.730184	2339.736832
	54	0.044914725	164.806855	8.02121	2447.019617
	55	0.122255763	153.639837	3.717309	2151.939199
	56	0.088766016	161.105793	3.742867	2193.943895
	57	0.08985357	160.973484	3.653504	2302.444041
	58	0.11004301	156.439179	3.704054	2328.918065
	59	/	/	3.02051	2429.457622
				Total length = 80.658666	
05	60	/	/	/	2382.660204
	61	0.127340889	153.063219	3.405036	2220.232687
	62	0.017864658	177.65071	3.910036	2131.49045
	63	0.074950773	161.104471	0.679631	2068.820561
	64	/	/	7.975904	1503.980946
				Total length = 15.970606	
06	65	/	/	/	1015.410161
	66	0.164080645	126.077703	7.078751	1729.905339
	67	0.301668309	136.307136	3.851418	1980.25674
	68	/	/	0.942993	2060.221926
				Total length = 11.873162	
07	69	/	/	/	144.396862
	70	0.174826997	93.634595	1.74601	418.480175
	71	0.15495261	92.427552	11.163383	788.332626
	72	0.173591205	92.074262	6.00008	1178.111817
	73	0.075420419	156.879709	9.612026	1324.298225
	74	0.057885399	173.6094	0.864513	1202.350599
	75	0.144025042	150.604976	2.985388	1291.147293
	76	0.184819859	136.472258	4.055459	1266.005016
	77	/	/	3.969197	1015.410161
				Total length = 40.396056	
08	78	/	/	/	608.420858
	79	0.271915245	88.919387	4.08322	635.014407
	80	0.316718066	92.165454	6.13259	574.111171
	81	/	/	1.354355	330.63154
				Total length = 11.570165	
09	82	/	/	/	99.419211
	83	0.153425766	90.134073	8.649979	1420.835847
	84	/	/	9.46424	533.904189
				Total length = 18.11422	
Maximum value		0.316718066	179.830519	12.674381	2447.019617
Minimum value		0.001114635	88.919387	0.64031	99.419211

**Table A14:** Master of the Nets Garden, corridor.

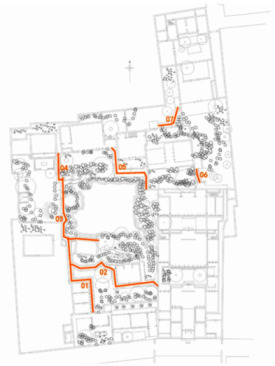

**Road No.**	**Angle No.**	**Curvatur**	**Value of Angle (°)**	**Unilateral Length of Path (m)**	**Viewshed Area (m^2^)**
01	0	/	/	/	936.488852
	1	0.098958712	161.648995	1.072485	855.622636
	2	0.000153759	179.933871	5.336088	267.610759
	3	0.166785827	89.999998	9.674613	327.728144
	4	0.14046187	89.967246	7.084926	679.998842
	5	/	/	12.354981	856.8409
				Total length = 35.523092	
02	6	/	/	/	393.457769
	7	0.271179789	148.068781	0.715965	366.510173
	8	0.114552909	150.110974	3.274681	385.694911
	9	0.135244526	145.740856	5.706755	749.913706
	10	0.547224594	92.800916	2.963257	975.082888
	11	0.255450979	147.25567	1.991969	1006.985225
	12	0.201265068	158.658242	2.420007	994.247893
	13	0.205973024	134.054017	1.253495	929.287521
	14	0.130141949	89.272999	6.048439	808.016662
	15	0.101053932	127.870401	14.203124	551.654141
	16	/	/	2.126163	608.305833
				Total length = 40.703855	
03	17	/	/	/	1056.596356
	18	0.044784565	159.140265	12.21199	808.2351
	19	0.414486051	120.398928	2.995968	910.481866
	20	0.196446011	141.678564	1.746474	914.757852
	21	0.295903434	109.926114	4.849143	911.797695
	22	0.201958294	152.858948	2.77393	922.004992
	23	0.042266732	167.143219	1.868264	953.266059
	24	/	/	8.699447	1298.121331
				Total length = 35.145217	
04	25	/	/	/	945.814098
	26	0.199269389	90.977906	9.814049	727.986807
	27	/	/	1.524055	1056.596356
				Total length = 11.338104	
05	28	/	/	/	406.931681
	29	0.139921086	154.335623	4.589627	1016.100965
	30	0.164390854	114.390051	1.586762	1007.82412
	31	0.14769054	93.274204	10.330472	1376.995592
	32	0.130357527	140.74587	8.151396	999.346571
	33	/	/	1.912531	1014.893174
				Total length = 26.570789	
06	34	/	/	/	95.123125
	35	0.081899497	167.535115	2.358179	164.70475
	36	/	/	2.943645	272.564352
				Total length = 5.301824	
07	37	/	/	/	185.501219
	38	0.188961062	145.704054	5.102781	236.159898
	39	0.183342938	145.932828	1.009376	236.598176
	40	0.026776485	175.525481	5.248204	237.354501
	41	/	/	0.580495	237.046774
				Total length = 11.940855	
Maximum value		0.547224594	179.933871	14.203124	1376.995592
Minimum value		0.000153759	89.272999	0.580495	95.123125

**Table A15:** Garden of Harmony, corridor.

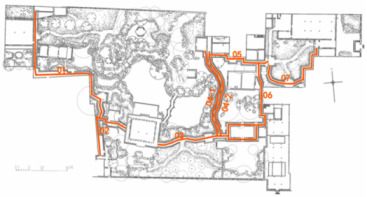

**Road No.**	**Angle No.**	**Curvature**	**Value of Angle (°)**	**Unilateral Length of Path (m)**	**Viewshed Area (m^2^)**
01	0	/	/	/	334.366723
	1	0.26728606	140.673129	2.523912	270.115148
	2	0.164857175	156.859268	2.511933	267.689563
	3	0.349306441	113.465937	2.355098	145.190102
	4	0.178474767	121.279177	3.849555	650.972491
	5	0.188326994	131.625893	6.995635	1770.1907
	6	0.064469784	137.98909	1.32586	1720.219104
	7	0.097988648	97.125291	19.757499	510.203482
	8	0.013665427	170.586219	2.631127	557.015521
	9	/	/	18.254936	25.80581
				Total length = 60.205554	
02	10	/	/	/	188.710065
	11	0.095402103	157.355617	3.216952	521.631955
	12	0.392105684	92.765593	4.927925	273.108631
	13	0.539911638	93.746464	1.077068	256.102642
	14	0.139629724	75.369205	3.466335	370.055657
	15	/	/	14.27787	249.646904
				Total length = 26.96615	
03	16	/	/	/	872.014366
	17	0.157528372	156.388462	2.533686	1377.826098
	18	0.039738528	173.888042	2.661201	1468.728226
	19	0.078618786	165.887126	2.705021	1524.611418
	20	0.10534999	159.491329	3.544585	1754.685284
	21	0.0706076	157.101587	3.214024	1747.885526
	22	0.031694863	168.585726	7.989415	478.102424
	23	/	/	4.556516	392.216026
				Total length = 27.204449	
04-1	24	/	/	/	578.6558
	25	0.097772535	173.575984	0.857401	1042.182881
	26	0.098014531	163.939683	2.470061	1189.814638
	27	0.142894763	153.791516	3.231855	1475.963901
	28	0.156734985	152.33125	3.114603	1529.716199
	29	0.110571565	159.628909	2.988142	1498.579168
	30	0.071184111	167.039585	3.40939	1519.148359
	31	0.192664683	149.273372	2.932748	1517.860953
	32	0.107667577	162.799175	2.566539	1351.489607
	33	0.139308495	159.658876	2.9323	1146.112508
	34	0.084567223	167.723723	2.231968	1086.626294
	35	0.170495617	151.213096	2.916654	1295.671726
	36	0.058270655	175.050386	2.999996	1178.27202
	37	0.231867594	169.07213	1.860286	965.427194
	38	/	/	0.197378	761.191414
				Total length = 34.709323	
04-2	39	/	/	/	90.32867
	40	0.150407834	175.885103	1.851065	498.137279
	41	0.068835064	153.024848	2.4564	297.664039
	42	0.141826156	166.726732	2.749123	204.300769
	43	0.188071326	158.088467	1.989338	161.001813
	44	0.100530826	163.432675	3.229399	252.469355
	45	0.055344059	150.793327	3.108109	331.081199
	46	0.160581066	169.460201	3.172309	246.498595
	47	0.091848006	161.478448	3.466137	379.802042
	48	0.141605493	150.760663	2.936268	307.906862
	49	0.09871789	157.782246	2.430446	307.889703
	50	0.16166287	167.415265	3.080556	295.581955
	51	0.056655748	169.442607	2.88987	510.059025
	52	/	/	0.782557	659.92195
				Total length = 34.141578	
05	53	/	/	/	242.490588
	54	0.120188552	146.603164	1.21726	332.003129
	55	0.244014137	92.106408	8.01806	304.020687
	56	0.316317318	89.785212	1.403782	321.210942
	57	0.311661891	94.146096	6.170202	642.171468
	58	0.266766925	96.741025	1.312456	459.589809
	59	/	/	6.560628	312.19754
				Total length = 24.682388	
06	60	/	/	/	400.384448
	61	0.089123395	159.556174	5.178042	282.617361
	62	0.05087567	158.157209	2.774117	361.670552
	63	0.165250724	90.000005	12.013767	473.85091
	64	0.520707635	90.000004	1.465052	343.6812
	65	0.140946323	90.011722	3.550547	18.555555
	66	0.135028186	89.893022	13.737657	21.929453
	67	0.168769468	89.763488	5.563233	63.490406
	68	0.168899807	90	10.486327	75.783419
	69	/	/	5.500457	154.667417
				Total length = 60.269198	
07	70	/	/	/	343.124332
	71	0.201622876	146.944657	3.286059	398.147636
	72	0.306226579	132.860892	2.315818	408.25993
	73	0.074066318	168.683972	2.900987	312.497402
	74	0.058678082	169.782793	2.423006	188.00786
	75	0.151283063	151.5483	3.646657	122.265531
	76	0.160200608	161.705747	2.847531	269.564398
	77	0.426811694	100.157456	1.111742	348.290101
	78	0.364963045	93.772276	4.284685	403.178613
	79	0.326663954	94.95919	3.127202	322.745645
	80	0.379966168	91.186908	4.973591	349.870588
	81	0.648475119	90.017047	1.619793	209.75423
	82	/	/	2.543079	269.234838
				Total length = 35.08015	
Maximum value		0.648475119	175.885103	19.757499	1770.1907
Minimum value		0.013665427	75.369205	0.197378	18.555555

**Table A16:** Lion Forest Garden, corridor.

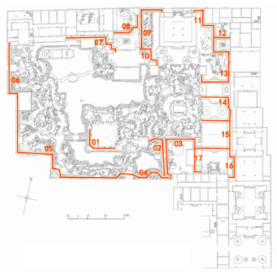

**Road No.**	**Angle No.**	**Curvature**	**Value of Angle (°)**	**Unilateral Length of Path (m)**	**Viewshed Area (m^2^)**
01	0	/	/	/	2095.597347
	1	0.156440832	127.084859	8.389355	2347.35256
	2	0.090074114	141.630911	2.636852	2216.840014
	3	/	/	11.617636	1787.258771
				Total length = 22.643842	
02	4	/	/	/	1768.939694
	5	0.096665882	98.679102	19.361663	982.912413
	6	0.146320486	147.173393	4.28828	797.173904
	7	/	/	3.43213	1064.465676
				Total length = 27.082073	
03	8	/	/	/	816.010218
	9	0.321038889	89.792344	3.918304	737.790885
	10	0.125267662	89.520177	4.85741	939.510285
	11	0.08561916	87.611773	15.249113	980.322939
	12	0.098866997	109.998113	18.315248	1757.985855
	13	0.554299108	107.130063	1.807557	1717.663729
	14	/	/	2.420232	1587.164025
				Total length = 46.567865	
04	15	/	/	/	684.43394
	16	0.114956501	154.48378	3.67103	925.28215
	17	0.205915635	133.007731	4.012726	1514.36019
	18	0.237113284	133.007731	3.730976	1851.920196
	19	0.241827121	131.442597	2.986924	1829.245318
	20	0.213364007	131.476778	3.804224	1707.418413
	21	/	/	3.898992	1672.443364
				Total length = 22.104872	
05	22	/	/	/	684.43394
	23	0.02534538	175.211816	4.779535	1149.491318
	24	0.331356282	133.062619	1.811834	758.11395
	25	0.046347917	132.362427	2.969318	352.860549
	26	0.067051263	89.528016	29.808466	119.183143
	27	0.119316141	166.469898	1.322732	75.723531
	28	0.398598148	119.90509	2.623367	60.03817
	29	0.134594224	165.778822	2.399748	63.741963
	30	0.263519582	94.260856	1.276336	651.965659
	31	0.097167771	94.532436	7.365971	1525.986603
	32	0.067479384	93.382988	18.577606	975.276911
	33	/	/	21.957301	433.419443
				Total length = 94.892215	
06	34	/	/	/	432.494849
	35	0.197001491	90.747109	9.129473	78.103551
	36	0.073625498	89.939001	4.321306	143.179434
	37	/	/	26.759224	91.096344
				Total length = 40.210003	
07	38	/	/	/	478.649341
	39	0.093201417	89.722334	20.695047	558.467182
	40	0.416612781	91.997304	3.714788	692.443801
	41	0.516107911	91.392835	2.909458	363.757203
	42	0.622304034	90.373318	2.488193	191.478999
	43	/	/	2.017906	132.106991
				Total length = 31.825392	
08	44	/	/	/	385.056189
	45	0.139214682	130.291932	8.289553	516.999248
	46	0.308848603	128.969683	3.499359	445.387913
	47	0.287820952	90.119066	2.035499	529.06691
	48	/	/	6.447267	344.875044
				Total length = 20.271677	
09	49	/	/	/	485.477156
	50	0.085499314	89.518148	20.768787	352.728556
	51	0.18210014	88.619882	10.937328	181.565034
	52	/	/	1.167421	30.669805
				Total length = 32.873536	
10	53	/	/	/	589.031552
	54	0.451197533	97.537734	2.75683	987.920882
	55	0.299165241	95.237234	2.989965	612.029924
	56	0.240583098	90.342624	5.68149	767.171566
	57	/	/	5.992665	419.951268
				Total length = 17.420949	
11	58	/	/	/	32.446
	59	0.425278048	93.66069	0.697388	44.335494
	60	0.221193354	86.223376	4.588439	157.91407
	61	0.200790821	89.884066	8.076425	270.058309
	62	/	/	5.846014	227.954354
				Total length = 19.208266	
12	63	/	/	/	383.551946
	64	0.184725622	91.242308	8.549832	365.405052
	65	/	/	6.289699	227.954354
				Total length = 14.839531	
13	66	/	/	/	365.405052
	67	0.1814569	88.696223	7.779244	406.062568
	68	0.113297107	88.219439	7.983033	417.950989
	69	0.092671053	90.894067	15.984937	237.162947
	70	0.136150965	89.65455	14.248881	163.180996
	71	/	/	3.546014	73.984368
				Total length = 49.542108	
14	72	/	/	/	309.493149
	73	0.128620494	91.656082	14.574568	290.522916
	74	0.1169354	155.178796	4.996188	287.977899
	75	0.12734891	151.597255	2.332011	293.34166
	76	0.125726277	91.678063	5.33617	294.814765
	77	/	/	14.702068	215.613066
				Total length = 41.941005	
15	78	/	/	/	294.814765
	79	0.098254659	89.310739	14.153094	292.416047
	80	/	/	14.798855	443.273243
				Total length = 28.951949	
16	81	/	/	/	443.273243
	82	0.390053643	95.139346	4.348736	403.09807
	83	0.179389579	95.157455	2.316097	329.440852
	84	0.179924343	89.981892	10.653339	214.269274
	85	/	/	3.176222	40.167106
				Total length = 20.494394	
17	86	/	/	/	1107.66515
	87	0.154037094	90.009214	3.480521	1325.997857
	88	/	/	3.009743	253.390746
				Total length = 6.490264	
Maximum value		0.622304034	175.211816	29.808466	2347.35256
Minimum value		0.02534538	86.223376	0.697388	30.669805

**Table A17:** Retreat and Reflection Garden, corridor.

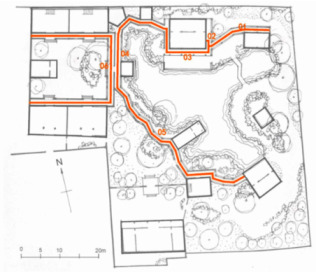

**Road No.**	**Angle No.**	**Curvature**	**Value of Angle (°)**	**Unilateral Length of Path (m)**	**Viewshed Area (m^2^)**
01	0	/	/	/	530.66
	1	0.02114709	168.29	6.469778	1218.95
	2	0.04139383	156.85	3.170261	1424.79
	3	/	/	6.499362	1506.26
				Total length = 16.139401	
02	4	/	/	/	1516.26
	5	0.63679680	90.00	0.751909	1448.17
	6	0.64315757	90.26	1.378664	1102.58
	7	/	/	0.6890795	1043.01
				Total length = 2.819653	
03	8	/	/	/	713.54
	9	0.16816696	90.00	0.7027335	681.75
	10	0.08114922	90.00	5.9048215	1500.30
	11	0.09152165	90.00	10.8161505	1528.73
	12	/	/	1.548118	1448.17
				Total length = 18.9718235	
04	13	/	/	/	713.54
	14	0.06485253	155.30	3.606894	949.02
	15	0.05845038	160.51	2.986539	1099.35
	16	0.16787187	117.18	2.8040855	941.44
	17	0.10297334	150.05	3.3940915	957.46
	18	0.01811593	164.29	1.601603	992.81
	19	/	/	13.397029	1266.52
				Total length = 27.7902425	
05	20	/	/		1266.52
	21	0.07973338	152.24	3.270702	1345.25
	22	0.06423891	158.32	2.744924	1331.93
	23	0.05369868	161.77	3.109678	1357.80
	24	0.09688209	147.70	2.7913545	1371.41
	25	0.07361419	153.75	2.9506125	1493.18
	26	0.11030726	144.62	3.218048	1463.26
	27	0.18775966	125.34	2.2830115	995.01
	28	0.05074630	163.75	2.604493	1010.40
	29	0.04410122	162.56	2.9653705	1113.08
	30	0.09358275	120.55	3.9097665	1073.99
	31	0.14954169	89.99	6.5770925	1496.73
	32	0.27195757	109.52	1.20903	1300.90
	33	0.03930289	167.57	2.8691825	1387.48
	34	0.09341505	147.99	2.6406205	1394.55
	35	/	/	3.233038	777.42
				Total length = 46.376925	
06	36	/	/	/	407.34
	37	0.03773271	89.59	20.4715355	408.93
	38	0.03712483	90.50	16.6764125	403.00
	39	/	/	20.9440315	419.05
				Total length = 58.0919795	
Maximum value		0.643157568	168.292461	20.9440315	1528.728816
Minimum value		0.018115933	87.878286	0.6890795	403.00353

**Table A18:** Couple’s Garden Retreat, corridor.

Part1		The East Garden of Couple’s Garden Retreat			

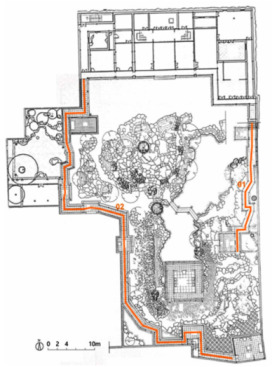

**Road No.**	**Angle No.**	**Curvature**	**Value of Angle (°)**	**Unilateral Length of Path (m)**	**Viewshed Area (m^2^)**
01	0	/	/	/	122.227443
	1	0.017621536	175.472183	5.0737	665.893765
	2	0.03588076	172.518002	3.865872	832.21308
	3	0.199982347	145.547956	3.406955	867.570446
	4	0.195993233	147.500275	2.509622	874.522799
	5	0.593934435	89.380713	3.198037	886.527023
	6	0.502829928	89.123129	1.089011	885.330502
	7	0.166665749	159.477279	0.781569	878.910707
	8	0.173173898	159.165048	3.465444	848.424104
	9	0.359442179	91.179637	0.679504	822.558166
	10	0.402531874	90.599662	1.79268	832.999669
	11	/	/	4.570159	90.269426
				Total length = 30.432553	
02	12	/	/	/	426.889145
	13	0.244015866	89.983436	6.989026	329.455519
	14	0.270042115	89.894799	4.168549	317.619887
	15	0.068843904	163.258081	6.129384	607.346234
	16	0.123572775	163.088753	2.309795	789.376938
	17	0.601353712	108.496897	2.449398	892.421418
	18	0.58783682	107.385149	1.356504	882.407925
	19	0.069188706	172.407681	2.572298	931.435437
	20	0.136169796	169.118588	1.253301	887.73562
	21	0.182564366	164.168063	1.530384	841.553974
	22	0.117416218	167.944068	1.486427	767.587459
	23	0.436545732	91.389823	2.090885	695.283244
	24	0.057128766	170.58614	4.024485	780.072499
	25	0.158278102	164.310636	1.718266	835.051785
	26	0.047530876	170.004579	1.730893	871.887413
	27	0.093263883	106.776919	5.593546	1101.042324
	28	0.050248948	143.885799	18.206378	829.983776
	29	0.124772264	145.698212	6.152627	669.896675
	30	0.396053801	109.561921	3.258944	623.034714
	31	0.304484588	90.306899	2.543544	510.696311
	32	0.319526136	86.768515	6.042299	253.240634
	33	0.504601741	87.272483	1.971609	397.292172
	34	0.345834736	89.911781	3.528239	486.914885
	35	0.223710441	99.272325	4.587544	369.794701
	36	/	/	6.83386	411.708229
				Total length = 98.528185	
**Part2**		**The West Garden of Couple’s Garden Retreat**			

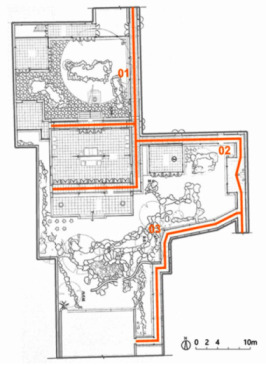

**Road No.**	**Angle No.**	**Curvature**	**Value of Angle (°)**	**Unilateral Length of Path (m)**	**Viewshed Area (m^2^)**
01	0	/	/	/	72.159114
	1	0.069166655	89.995593	25.903041	351.751389
	2	/	/	12.67904	301.585628
				Total length = 38.582081	
02	3	/	/	/	145.579924
	4	0.125522503	89.490435	15.727921	243.844503
	5	0.100164425	164.660589	2.69012	313.155995
	6	0.205082814	148.578236	2.640112	337.874407
	7	0.063358104	164.562554	2.64134	342.21415
	8	/	/	5.797079	71.395212
				Total length = 29.496573	
03	9	/	/	/	62.213071
	10	0.181892611	90.151321	3.243991	69.281611
	11	0.188859914	91.237904	10.47756	89.911215
	12	0.520661897	91.065678	1.311372	136.426937
	13	0.138922697	160.149947	3.585511	239.754595
	14	0.21362043	119.612124	1.362876	282.998958
	15	0.04716916	165.123696	7.379103	305.12638
	16	0.19817947	155.312234	3.587357	323.467248
	17	/	/	0.68109	323.811811
				Total length = 31.62886	
Maximum value		0.520661897	165.123696	25.903041	351.751389
Minimum value		0.04716916	89.490435	0.68109	62.213071

**Table A19:** Jichang Garden, corridor.

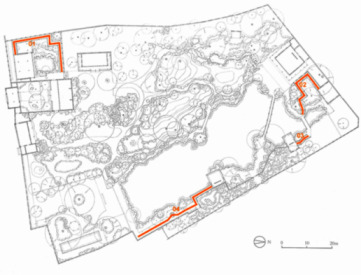

**Road No.**	**Angle No.**	**Curvature**	**Value of Angle (°)**	**Unilateral Length of Path (m)**	**Viewshed Area (m^2^)**
01	0	/	/	/	373.213118
	1	0.164388538	89.40132	11.52946	1889.466068
	2	0.406271004	86.217017	3.858941	1584.781305
	3	0.137936823	82.085717	3.304533	1698.201863
	4	0.129416986	91.680462	14.438405	179.414965
	5	/	/	4.953729	8.221111
				Total length = 38.085069	
02	6	/	/	/	2754.289322
	7	0.212099257	90.000004	7.368593	2787.550633
	8	0.234332304	93.163557	5.781068	3625.158237
	9	0.301911661	92.54829	5.950262	3500.198804
	10	0.323369019	107.565528	2.644068	3585.981425
	11	0.133121819	155.862097	4.532405	3644.391825
	12	/	/	1.721333	3591.615757
				Total length = 27.997729	
03	13	/	/	/	1919.602632
	14	0.129842146	163.676072	1.792436	1766.111212
	15	0.292715269	146.264482	2.580212	1247.306883
	16	/	/	1.292419	908.430535
				Total length = 5.665066	
04	17	/	/	/	3523.856384
	18	0.010768353	172.96856	19.835953	3204.075103
	19	0.143575126	90.695255	2.918882	3059.918797
	20	0.047508198	157.724277	13.584311	2905.885936
	21	0.328774617	132.32379	2.532721	2913.632062
	22	0.153150524	153.189377	2.383742	2843.814741
	23	/	/	3.663526	2740.065405
				Total length = 44.919135	1569.488678
Maximum value		0.406271004	163.676072	19.835953	3644.391825
Minimum value		0.129416986	82.085717	1.292419	8.221111

**Table A20:** Humble Administrator’s Garden, bridge.

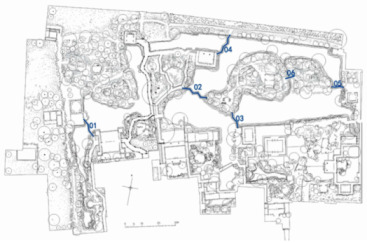

**Road No.**	**Angle No.**	**Curvature**	**Value of Angle (°)**	**Unilateral Length of Path (m)**	**Viewshed Area (m^2^)**
01	0	/	/	/	3722.328935
	1	0.158519754	149.616274	3.989135	4240.760713
	2	0.134737411	149.250694	3.77733	4388.367995
	3	/	/	4.093192	2431.119518
				Total length = 11.859657	
02	4	/	/	/	8981.878518
	5	0.205161258	134.139524	4.578015	9082.299147
	6	0.318397391	123.172386	2.988332	8897.820649
	7	0.342006326	118.965893	2.98956	8989.166356
	8	0.263281108	128.037711	2.949403	8773.851019
	9	/	/	3.696266	8424.518842
				Total length = 17.201576	
03	10	/	/	/	8816.411375
	11	0.1739709	146.590771	2.995085	8622.898065
	12	0.158431111	147.924787	3.611197	8801.479115
	13	/	/	3.363538	8113.410698
				Total length = 9.96982	
04	14	/	/	/	1841.560527
	15	0.116464527	130.093335	3.025697	3106.009257
	16	0.193989718	146.948782	3.206419	3907.973409
	17	0.285618473	132.373919	3.259382	4161.618193
	18	0.087993382	146.178496	3.121515	4210.883244
	19	0.210740835	174.767901	1.727308	4363.938616
	20	/	/	0.485206	4420.438654
				Total length = 14.825527	
05	21	/	/	/	6124.714272
	22	0.039467993	157.253993	1.83974	6417.46891
	23	0.367986233	154.701178	3.024955	5611.345352
	24	/	/	2.369764	5798.420764
				Total length = 7.234459	
06	25	/	/	/	6321.750442
	26	0.031050722	175.103352	1.955501	6333.722451
	27	/	/	3.547102	6490.965533
				Total length = 5.502603	
Maximum value		0.367986233	175.103352	4.578015	9082.299147
Minimum value		0.031050722	118.965893	0.485206	1841.560527

**Table A21:** Lingering Garden, bridge.

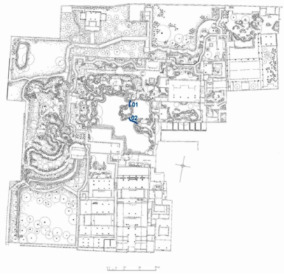

**Road No.**	**Angle No.**	**Curvature**	**Value of Angle (°)**	**Unilateral Length of Path (m)**	**Viewshed Area (m^2^)**
01	0	/	/	/	2129.081722
	1	0.198268364	155.456131	2.30644	2137.78113
	2	/	/	1.981141	2162.315865
				Total length = 4.287581	
02	3	/	/	/	2213.3978
	4	0.296387302	138.777677	2.103434	2240.850596
	5	0.134160566	161.537099	2.643094	2219.122421
	6	/	/	2.139222	2214.834308
				Total length = 6.885751	
Maximum value		0.296387302	161.537099	2.643094	2240.850596
Minimum value		0.134160566	138.777677	1.981141	2129.081722

**Table A22:** Canglang Pavilion, bridge.

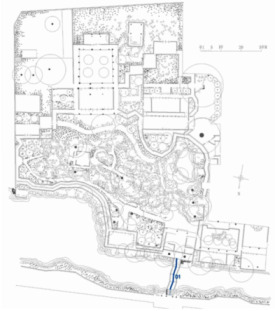

**Road No.**	**Angle No.**	**Curvature**	**Value of Angle (°)**	**Unilateral Length of Path (m)**	**Viewshed Area (m^2^)**
01	0	/	/	/	2856.891253
	1	0.05879935	164.525512	2.770826	2786.624414
	2	0.097450041	159.98639	4.130918	2664.989262
	3	0.09015669	158.698048	3.577943	2571.182451
	4	0.07799897	166.278582	4.008111	2412.35146
	5	/	/	4.118221	2309.824214
				Total length = 18.60602	
Maximum value		0.097450041	166.278582	4.130918	2856.891253
Minimum value		0.05879935	158.698048	2.770826	2309.824214

**Table A23:** Master of the Nets Garden, bridge.

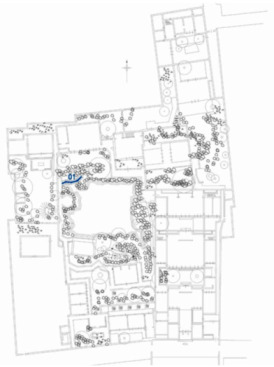

**Road No.**	**Angle No.**	**Curvature**	**Value of Angle (°)**	**Unilateral Length of Path (m)**	**Viewshed Area (m^2^)**
01	0	/	/	/	1241.671303
	1	0.228257431	149.798725	2.120106	1228.872906
	2	0.164858711	158.480635	2.444338	1151.638424
	3	0.306598622	147.789042	2.084854	1073.347403
	4	/	/	1.53076	909.809115
				Total length = 8.180058	
Maximum value		0.306598622	158.480635	2.444338	1241.671303
Minimum value		0.164858711	147.789042	1.53076	909.809115

**Table A24:** Garden of Harmony, bridge.

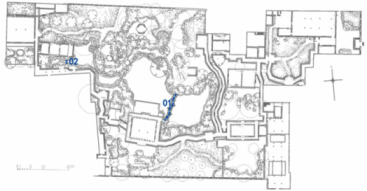

**Road No.**	**Angle No.**	**Curvature**	**Value of Angle (°)**	**Unilateral Length of Path (m)**	**Viewshed Area (m^2^)**
01	0	/	/	/	1397.218074
	1	0.249869305	143.54363	3.071704	1869.85599
	2	0.361806594	139.337049	1.921579	1875.63662
	3	0.34671281	139.337051	1.920021	1840.151468
	4	0.246696102	147.935408	2.088174	1822.466171
	5	/	/	2.3889	1799.105567
				Total length = 11.39037	
02	/	Straight	/	/	994.710509
				1.534972	1065.32671
Maximum value		0.361806594	147.935408	3.071704	1875.63662
Minimum value		0.246696102	139.337049	1.920021	1397.218074

**Table A25:** Lion Forest Garden, bridge.

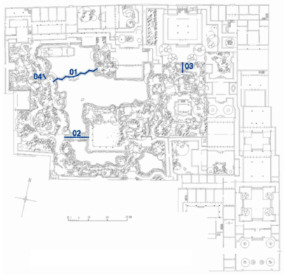

**Road No.**	**Angle No.**	**Curvature**	**Value of Angle (°)**	**Unilateral Length of Path (m)**	**Viewshed Area (m^2^)**
01	0	/	/	/	2396.741293
	1	0.334780914	128.761062	2.810267	2396.453275
	2	0.374123827	126.107807	2.351313	2355.327733
	3	0.406812445	131.551157	2.493093	2334.119797
	4	0.07698826	171.469741	1.517359	2343.434897
	5	0.145995135	159.656441	2.346222	2319.427099
	6	0.143627481	162.293503	2.49222	2378.754616
	7	0.399995172	132.572595	1.792589	2275.950028
	8	0.323082415	132.956559	2.224643	2241.576877
	9	0.334775232	131.334988	2.711916	2200.704619
	10	0.325723336	135.043491	2.205844	2165.287668
				2.487889	2140.66765
				Total length = 25.433354	
02	/	Straight	/	/	2934.01686
				12.562273	2517.691661
03	/	Straight	/	/	529.152657
				5.310536	490.19745
04	/	Straight	/	/	2404.116217
				2.534107	2443.47372
Maximum value		0.406812445	171.469741	12.562273	2396.741293
Minimum value		0.07698826	126.107807	1.517359	2140.66765

**Table A26:** Retreat and Reflection Garden, bridge.

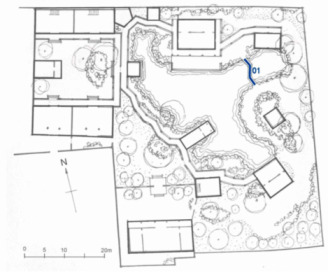

**Road No.**	**Angle No.**	**Curvature**	**Value of Angle (°)**	**Unilateral Length of Path (m)**	**Viewshed Area (m^2^)**
01	0	/	/	/	1796.02
	1	0.144795303	136.948404	2.151369	1856.15
	2	0.122811538	142.490057	2.908065	1851.65
	3	/	/	2.3241445	1953.55
				Total length = 7.3835785	
Maximum value		0.144795303	142.490057	2.908065	1953.548509
Minimum value		0.122811538	136.948404	2.151369	1796.01998

**Table A27:** Couple’s Garden Retreat, bridge.

Part1		The East Garden of Couple’s Garden Retreat			

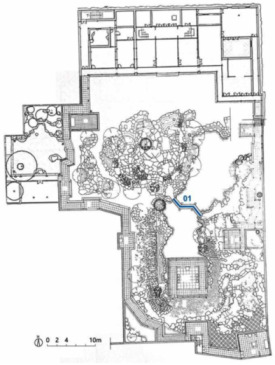

**Road No.**	**Angle No.**	**Curvature**	**Value of Angle (°)**	**Unilateral Length of Path (m)**	**Viewshed Area (m^2^)**
01	0	/	/	/	1078.271105
	1	0.344939517	129.9046	2.154972	1128.830327
	2	0.336914878	127.290767	2.746817	1123.803506
	3	/	/	2.522606	1144.077125
				Total length = 7.424395	
Part2		The west garden of Couple’s Garden Retreat (no bridge)			
Maximum value		0.344939517	129.9046	2.746817	1144.077125
Minimum value		0.336914878	127.290767	2.154972	1078.271105

**Table A28:** Jichang Garden, bridge.

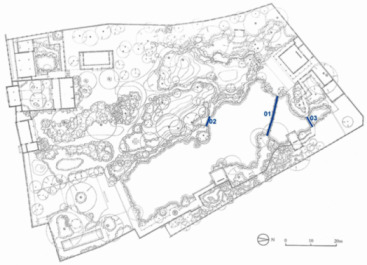

**Road No.**	**Angle No.**	**Curvature**	**Value of Angle (°)**	**Unilateral Length of Path (m)**	**Viewshed Area (m^2^)**
01	0	/	/	/	2911.726219
	1	0.011054088	154.791331	9.292266	2756.704503
	2	/	/	7.148453	2711.36196
				Total length = 16.440718	
02	/	Straight	/	/	3381.422258
				4.183686	3417.438274
03	/	Straight	/	/	3545.39741
				4.570671	2682.586158
Maximum value		0.011054088	154.791331	9.292266	3545.39741
Minimum value		0.011054088	154.791331	4.183686	2682.586158

## References

[1] Atwa S.M.H., Ibrahim M.G., Saleh A.M., Murata R. (2019). Development of sustainable landscape design guidelines for a green business park using virtual reality. Sustain. Cities Soc..

[2] French J.S. (1973). Urban Green: City Parks of the Western World.

[3] Heckscher A., Robinson P.C., Fledderus D. (1977). Open Spaces: The Life of American Cities.

[4] Turner T. (1992). Open space planning in London: From standards per 1000 to green strategy. Town Plan. Rev..

[5] Tibbetts J., Fausold C.J. (1998). Open Space Conservation.

[6] Feng L., Mi X., Yuan D. (2022). Optimal planning of urban greening system in response to urban microenvironments in a high-density city using genetic algorithm: A case study of Tianjin. Sustain. Cities Soc..

[7] Liu H., Hamel P., Tardieu L., Remme R.P., Han B., Ren H. (2022). A geospatial model of nature-based recreation for urban planning: Case study of Paris, France. Land Use Policy.

[8] He M., Wang Y., Wang W.J., Xie Z. (2022). Therapeutic plant landscape design of urban forest parks based on the Five Senses Theory: A case study of Stanley Park in Canada. Int. J. Geoherit. Parks.

[9] Seo S., Choi S., Kim K., Kim S.M., Park S.M. (2019). Association between urban green space and the risk of cardiovascular disease: A longitudinal study in seven Korean metropolitan areas. Environ. Int..

[10] Ma X., Tian Y., Du M., Hong B., Lin B. (2021). How to design comfortable open spaces for the elderly? Implications of their thermal perceptions in an urban park. Sci. Total Environ..

[11] D’Alessandro D., Buffoli M., Capasso L., Fara G.M., Rebecchi A., Capolongo S. (2015). Green areas and public health: Improving wellbeing and physical activity in the urban context. Epidemiol. E Prev..

[12] Nieuwenhuijsen M.J. (2021). New urban models for more sustainable, liveable and healthier cities post covid19; reducing air pollution, noise and heat island effects and increasing green space and physical activity. Environ. Int..

[13] Hino A.A.F., Reis R.S., Ribeiro I.C., Parra D.C., Brownson R.C., Fermino R.C. (2010). Using observational methods to evaluate public open spaces and physical activity in Brazil. J. Phys. Act. Health.

[14] Zhang J., Yu Z., Zhao B., Sun R., Vejre H. (2020). Links between green space and public health: A bibliometric review of global research trends and future prospects from 1901 to 2019. Environ. Res. Lett..

[15] Takano T., Nakamura K., Watanabe M. (2002). Urban residential environments and senior citizens’ longevity in megacity areas: The importance of walkable green spaces. J. Epidemiol. Community Health.

[16] Ji C. (1988). Craft of Gardens.

[17] Jun T. (2014). Jiangnan yuanlin zhi. History of Gardens in Jiangnan.

[18] Zhao J. (2008). Analysis of Lingering Garden Space Based on Visual Perception. Master’s Thesis.

[19] Yan Y. (2009). Detour and Entry: The Influence of Path on Experience Density in Private Gardens of Ming and Qing Dynasties in Suzhou. Master’s Thesis.

[20] Cai L. On the Application of Horizon Analysis Theory in the Study of Chinese Classical Gardens—Taking the Entrance Space Sequence of Lingering Garden as an Example. Proceedings of the 2009 Annual Conference of the Chinese Society of Landscape Architecture.

[21] Jin Y. (2011). Quantitative Research on Path Space of Private Gardens in the Jiangnan. Master’s Thesis.

[22] Xu D. (2013). Comparative Study and Application of the Meandering Path Pattern and Maze Pattern in Chinese and Western Gardens. Master’s Thesis.

[23] Zhang Y. (2015). Quantitative Research on Jiangnan Private Garden Space Based on Line of Sight. Master’s Thesis.

[24] Lu P. (2017). Research on the Law of Stagnation Points in the Spatial Path of the. Master of the Nets Garden Based on Video Analysis Technology. Master’s Thesis.

[25] Niu Y. (2015). Research on the Verification of the “Garden” Space of Suzhou Lingering Garden Based on Digital Technology. Master’s Thesis.

[26] Yu S. (2022). Simulation and Application of Urban Road Landscape Based on Geographic Information Data. J. Sens..

[27] Levus Y.V., Morozov M.Y., Moravskyi R.O., Pustelnyk P.Y. (2022). Algorithms And Architecture Of The Software System Of Automated Natural And Anthropogenic Landscape Generation. Radio Electron. Comput. Sci. Control.

[28] Monedero J. (2000). Parametric design: A review and some experiences. Autom. Constr..

[29] Lee J.H., Ostwald M.J. (2020). Creative Decision-Making Processes in Parametric Design. Buildings.

[30] Hernandez C.R. (2006). Thinking parametric design: Introducing parametric Gaudi. Des. Stud..

[31] Burry M. (1996). Parametric design and the Sagrada Familia. Arq Archit. Res. Q..

[32] Havemann S., Fellner D. (2004). Generative parametric design of gothic window tracery. Proceedings Shape Modeling Applications.

[33] Kabošová L., Katunský D., Kmet S. (2020). Wind-based parametric design in the changing climate. Appl. Sci..

[34] Bande L., Alshamsi A., Alhefeiti A., Alderei S., Shaban S., Albattah M., Scoppa M.D. (2021). Parametric design structures in low rise buildings in relation to the urban context in UAE. Sustainability.

[35] ElBatran R.M., Ismaeel W.S. (2021). Applying a parametric design approach for optimizing daylighting and visual comfort in office buildings. Ain Shams Eng. J..

[36] Wang Y., Chen L. (2022). Architectural and landscape garden planning integrated with artificial intelligence parametric analysis. Secur. Commun. Netw..

[37] Guo S., Tang J., Liu H., Gu X. (2021). Study on landscape architecture model design based on big data intelligence. Big Data Res..

[38] Marafa L.M., Wang Z., Tsang F.K. (2022). Tranquillity in urban classical Chinese gardens and modern parks: The effect of natural and contextual features. Sustainability.

[39] Fung S. (1999). Here and there in Yuan ye. Stud. Hist. Gard. Des. Landsc..

[40] Xu Z., Gui W., Heidari A.A., Liang G., Chen H., Wu C., Turabieh H., Mafarja M. (2021). Spiral motion mode embedded grasshopper optimization algorithm: Design and analysis. IEEE Access.

[41] Arzamendia M., Espartza I., Reina D.G., Toral S.L., Gregor D. (2019). Comparison of eulerian and hamiltonian circuits for evolutionary-based path planning of an autonomous surface vehicle for monitoring ypacarai lake. J. Ambient. Intell. Humaniz. Comput..

[42] Liang H., Li W., Lai S., Jiang W., Zhu L., Zhang Q. (2020). How to survey, model, and measure rockeries in a Chinese classical garden: A case study for Huanxiu Shanzhuang, Suzhou, China. Landsc. Res..

[43] Liu M., Li Y., Li A., Huo Q., Zhang N., Qu N., Zhu M., Chen L. (2020). A slime mold-ant colony fusion algorithm for solving traveling salesman problem. IEEE Access.

[44] Mirjalili S.Z., Mirjalili S., Saremi S., Faris H., Aljarah I. (2018). Grasshopper optimization algorithm for multi-objective optimization problems. Appl. Intell..

[45] Li X., Xia B., Lusk A., Liu X., Lu N. (2019). The Humanmade Paradise: Exploring the Perceived Dimensions and Their Associations with Aesthetic Pleasure for Liu Yuan, a Chinese Classical Garden. Sustainability.

[46] Li S., Chen H., Wang M., Heidari A.A., Mirjalili S. (2020). Slime mould algorithm: A new method for stochastic optimization. Future Gener. Comput. Syst..

